# CRISPR-Based Editing Techniques for Genetic Manipulation of Primary T Cells

**DOI:** 10.3390/mps3040079

**Published:** 2020-11-18

**Authors:** Mateusz Kotowski, Sumana Sharma

**Affiliations:** MRC Human Immunology Unit, John Radcliffe Hospital, University of Oxford, Oxford OX3 9DS, UK; mateusz.kotowski@rdm.ox.ac.uk

**Keywords:** primary T cells, CRISPR/Cas9, genome-editing, CAR-T cells

## Abstract

While clustered regularly interspaced short palindromic repeats (CRISPR)-based genome editing techniques have been widely adapted for use in immortalised immune cells, efficient manipulation of primary T cells has proved to be more challenging. Nonetheless, the rapid expansion of the CRISPR toolbox accompanied by the development of techniques for delivery of CRISPR components into primary T cells now affords the possibility to genetically manipulate primary T cells both with precision and at scale. Here, we review the key features of the techniques for primary T cell editing and discuss how the new generation of CRISPR-based tools may advance genetic engineering of these immune cells. This improved ability to genetically manipulate primary T cells will further enhance our fundamental understanding of cellular signalling and transcriptional networks in T cells and more importantly has the potential to revolutionise T cell-based therapies.

## 1. Introduction

In recent years, clustered regularly interspaced short palindromic repeats (CRISPR)/CRISPR-associated protein 9 (Cas9)-based techniques have transformed our ability to genetically manipulate mammalian genomes. Among the many fields within biomedical research to benefit from the ease of genetic manipulation using the CRISPR/Cas9 system is the study of the immune system and especially T cells. As T cells are the central regulators of the adaptive immune system and play a central role in many disease contexts such as cancer, infectious diseases and autoimmunity, elucidating the cellular physiology of these cells is of both basic biology and of clinical interest [1]. T cells engineered using the CRISPR/Cas9 system to increase their antitumor potency and reduce alloreactivity are already showing great promise in the creation of effective next generation adoptive cell therapies. Additionally, systematic studies using the CRISPR-based genome-scale editing approach form the basis of unbiased studies that shed light on fundamental T cell biology, thereby further enabling the design of better therapeutics.

**Figure 1:**
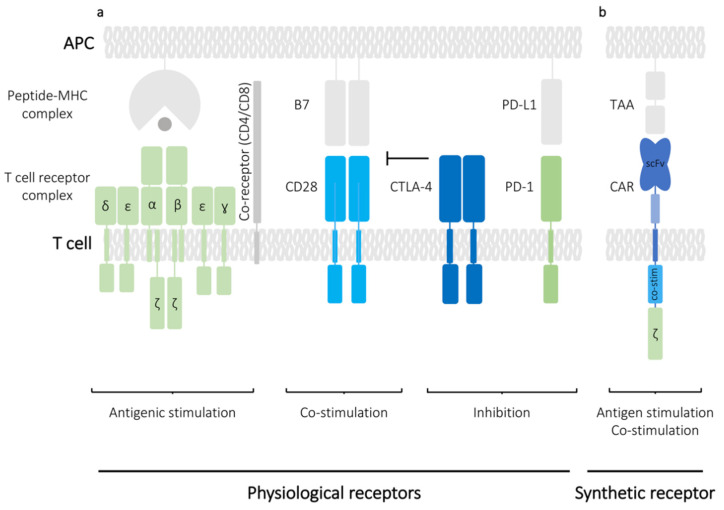
Overview of the different types of receptors present on the surface of T cells and their interaction partners. (**a**) The T cell receptor (TCR) complex is composed of eight polypeptide chains: αβγδ2ε2ζ. The hypervariable loops located at the top of the αβ dimer are responsible for recognition of antigenic peptides presented by major histocompatibility complex (MHC) I or II on the surface of an antigen-presenting cell (APC). Upon interactions of peptide-MHC with the TCR, a co-receptor binds to the side of the MHC. Co-receptors CD8 and CD4 interact exclusively with either MHCI or MHCII, respectively, and mature T cells express only one of the two co-receptor types. Interactions of co-stimulatory receptor CD28 with B7 are required for full activation of the T cell and can be inhibited by CTLA-4 which competes with CD28 for B7 binding. Another inhibitory receptor on the T cell surface is PD-1 which is triggered upon by binding to PD-L1 on the apposed cell. (**b**) Chimeric antigen receptors (CAR) permit the T cell response to be redirected towards a selected surface antigen, for example a tumour-associated antigens (TAA). The extracellular domain of CARs relies on a single chain variable fragment (scFv) for antigen recognition. Most commonly, the signalling chains of CARs consist of intracellular domains derived from co-stimulatory receptors (co-stim) and the ζ chain of the TCR.

**Figure 2:**
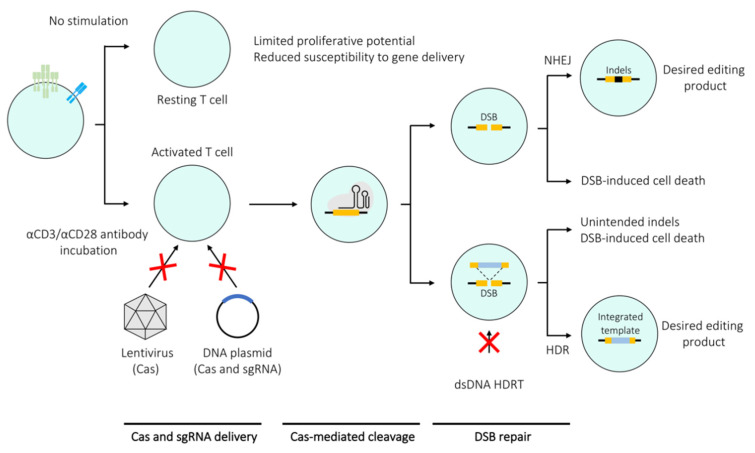
Barriers to editing of primary T cells. There are a number of technical difficulties that contributed to the delayed start of clustered regularly interspaced short palindromic repeats (CRISPR)-based editing of primary T cells. Unstimulated T cells are more difficult to edit due to their limited proliferative potential and reduced susceptibility to gene delivery. Lentiviral delivery of Cas-encoding genes into T cells is feasible, albeit very low editing efficiencies are achieved via this method. Presence of various DNA immunosensors in T cells means that bare double-stranded DNA (dsDNA) serves poorly as a vector for CRISPR components and homology directed repair templates (HDRT). The double-strand breaks (DSB) induced by Cas nucleases are associated with cellular toxicity and may eventually lead to cell death. Due to the competition between the non-homologous end joining (NHEJ) and homology directed repair (HDR) pathways, the HDR-based knockin may lead to unintended indels in a proportion of edited cells.

The transition of T cells from their resting to an activated state underpins most functions of adaptive immune responses. The initial signalling event in T cells occurs when the T cell receptors (TCR) expressed on the surface of both CD4^+^ helper T cells and CD8^+^ cytotoxic T cells interacts with their cognate antigenic peptides presented on major histocompatibility complex (MHC) molecules (Figure 1a). The TCR is a surface protein complex consisting of two different polypeptide chains with α and β chains forming the predominant type of TCR found in humans. The TCRα and TCRβ chains are encoded by *TRA* and *TRB* genes, respectively, which can be subdivided into a variable and a constant region signified by appending the gene name with either ‘*V/D/J*’ or ‘*C*’. The latter region is of particular importance for genetic manipulation of T cells since its sequence is identical for most T cells isolated from an individual donor, thus facilitating the reprogramming of antigen specificity. While the TCRαβ dimer is sufficient to recognise peptide antigens, it does not have its own signalling activity and instead associates with CD3 signalling co-receptors: CD3γ, CD3δ, two CD3ε and two CD3ζ (Figure 1a). MHC molecules can be subdivided into MHC class I (MHCI) and MHC class II (MHCII) recognised by CD8^+^ and CD4^+^ T cells, respectively. MHCI molecule is composed of non-covalently associated transmembrane α heavy chain and a globular β-2 microglobulin (β2M), whereas MHCII molecule consist of two transmembrane chains: α and β. In the classical paradigm of T cell signalling, the initial signalling event from the TCR-CD3 complex upon engagement with the MHC molecules is referred to as ‘signal one’. Full activation of T cells however requires a secondary signalling event called ‘signal two’, which is provided by a cell surface receptor CD28, which binds to its cognate ligand on the surface of an antigen-presenting cell (APC). In vitro, these signals can be mimicked by incubation of T cells with antibodies against CD28 and CD3 (αCD28/αCD3) immobilized on a glass plate or with αCD28/αCD3-coated beads (Figure 2) [2]. The surface of a T cell is also populated with a number of other receptors called immune checkpoints. These receptors modulate the T cell response by providing co-stimulatory or inhibitory signals. PD-1 and CTLA-4 are classic examples of inhibitory receptors and in recent years they have received considerable attention because blocking their functions using monoclonal antibodies can be successfully used to increase T cell responses against various cancers (Figure 1a) [3]. The intracellular components that govern the signalling event post-engagement of the TCR and signal modulation by the inhibitory receptors have been of interest to researchers for many years and the improvement in genetic editing of T cells has directly contributed to enriching our understanding of their biology.

The use of T cells in adoptive therapies has been a therapeutically lucrative field and is best exemplified by their applicability in cancer immunotherapy, which has recently gained widespread interest [4]. Cell-based immunotherapy relies on the genetic engineering to introduce synthetic transgenes into T cells, and nowhere has this been investigated more than in chimeric antigen receptor (CAR)-based immunotherapies. CAR T cells are T cells that have been genetically engineered to express an antigen recognition domain, which usually is a single chain variable fragment (scFv) recognizing a tumour associated antigen (TAA), fused to an intracellular CD3ζ chain and motifs from costimulatory proteins such as CD28 or CD137 that together mimic the signalling from the TCR and the co-stimulatory receptors (Figure 1b). Reliance on scFv for antigen binding allows the genetically engineered CAR T cells to generate signalling responses independent of the MHC molecules. CAR T cell-based therapies are revolutionizing treatment of multiple classes of leukemias and lymphomas, however their use in treatment of solid tumours is still limited [5]. As the techniques for genetic manipulation of T cells have become more advanced, much effort has been put into improving CAR T cell designs through gene modifications for enhanced anti-tumour activity.

**Figure 3:**
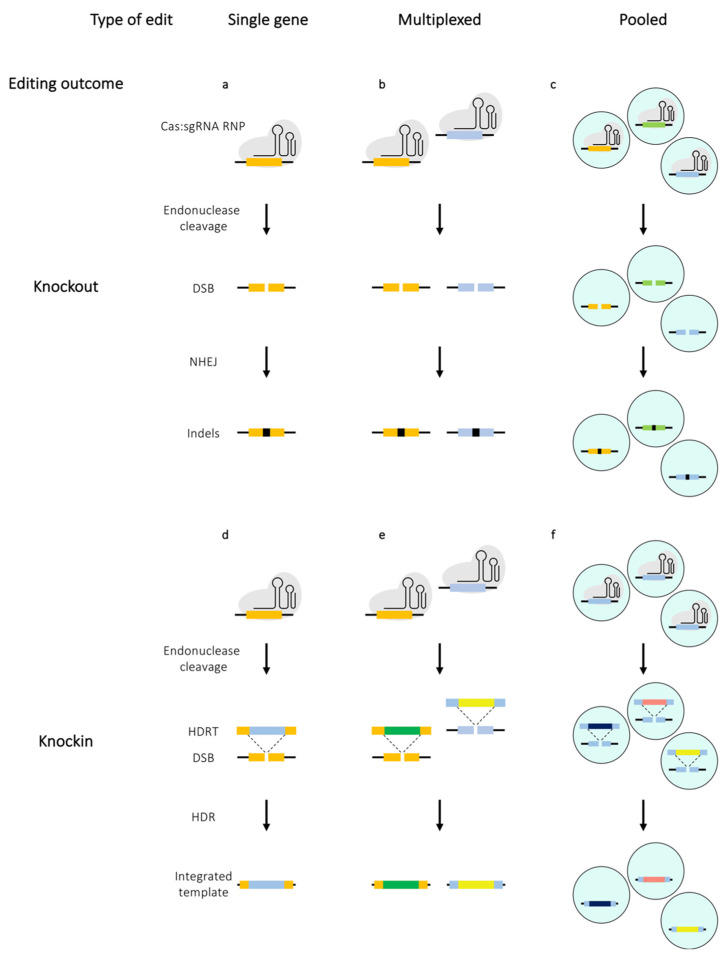
Summary of the most common strategies for genome manipulation in primary T cells. In terms of the resulting phenotypes, outcomes of CRISPR-based editing can be divided into knockouts (**a**–**c**) and knockins (**d**–**f**). The former results in abolition of the expression of the target gene and relies on non-homologous end joining (NHEJ) DNA repair pathway to instal indels. The latter can either result in alterations of the endogenous sequence at the target locus or an insertion of an exogenous sequence into the target locus. The new sequence is integrated via homology directed repair (HDR) and its identity is specified by an HDR template (HDRT). Both HDR and NHEJ pathways are initated by double-strand breaks (DSB). With regard to the number of loci edited per cell and the number of loci targeted in the cell population as a whole, editing strategies can be broadly divided into three types. (**a**,**d**) Single gene editing involves a genetic alteration at a single locus per cell. (**b**,**e**) Multiplexed editing results in modifications at multiple loci per cell. (**c**) The pooled knockouts aim to disrupt one locus per cell on average, however a large number of loci can be targeted in the edited population with the entire genome being screened in the extreme. (**f**) The pooled knockins involve the same locus being targeted in every cell, however the identity of the genetic modification at this locus differs between the cells in the edited population. Following a pooled knockin, each cell should only have a single species of HDRT integrated into the target locus on average.

Before the advent of the CRISPR/Cas9 era, genetic manipulations of T cells were performed mostly with other genome editing nucleases such as zinc-finger nucleases (ZFNs) and transcription activator-like effector nucleases (TALEN) [6,7,8]. While broadly successful, these approaches were mainly limited by their low efficiency and complex molecular cloning or substantial protein engineering required for targeting genomic sites [9]. The CRISPR/Cas9 system provides a convenient platform for targeting genomic loci with ease. The CRISPR/Cas9 technology is derived from the type II CRISPR/Cas system of the bacterial adaptive immune system, which utilises crRNA (CRISPR RNA), tracrRNA (trans-activating crRNA2) and a single large multi-domain effector protein (Cas9) to mediate both target recognition and cleavage. Subsequently, it was shown that the tracrRNA:crRNA duplex can be engineered as a single piece of chimeric RNA known as the single guide RNA (sgRNA) and by altering the sequences of the sgRNA, Cas9 endonuclease could be directed to a specific genomic locus to induce double-strand breaks (DSB) [10]. Such breaks are typically repaired by the cellular non-homologous end joining (NHEJ) DNA repair mechanism and this error-prone mechanism often results in random insertions and deletions (indels) at the site of the DSB, which can lead to a frameshift, the consequence of which can be either a premature STOP codon (pmSTOP) or an open reading frame (ORF) encoding a dysfunctional protein that is likely to be degraded shortly after translation (Figure 3a–c). In presence of a homologous sequence in the edited cell, the Cas-induced DSBs can be repaired via a homology directed repair (HDR) pathway (Figure 3d–f). In contrast to NHEJ, the outcome of HDR can be controlled by providing the edited cells with an appropriately designed homology directed repair template (HDRT). This generally permits alteration of the endogenous sequence or insertion of an exogenous sequence at the target locus.

Over the years, a number of improvements to Cas-based editing systems have been made. Endonuclease dead versions of Cas9 (dCas9) fused to transcription activator or repressor domains to create programmable transcriptional activators and repressors, respectively, are used to genetically program cells without creating DSBs [11,12]. More recently, Cas-based base editors are being used for precise genetic manipulations [13]. These enzymes are engineered by fusion of Cas9 nickase—a mutant version of Cas9 that only creates a single-strand break rather than DSBs—to either an adenine or a cytosine deaminase domain, which can be used to induce knockouts by mutation of splice sites or insertion of pmSTOPs into the target gene. A large amount of effort is continuously being put into expanding the CRISPR toolbox with the aim of increasing gene editing efficiency and precision [13,14].

The success of manipulating primary T cells using CRISPR systems however had a delayed start compared to other cell types and required substantial optimisation. Earlier studies demonstrated that CRISPR/Cas9 gene disruption in primary T cells using lentiviral and adenoviral vectors was challenging, even though such delivery systems had been highly successful in immortalised cell lines [15,16]. Despite the initial barriers to genetic manipulation of primary T cells, the expansion of Cas-based enzymes and delivery systems have now permitted these cells to be edited with both high accuracy and efficiency. The diverse assortment of CRISPR tools means that there are multiple considerations to be made prior to the manipulation. Over the years, a number of critical variables have been identified as being important in determining success of an editing experiment; these mainly include (i) type of Cas enzyme, sgRNA and optionally the HDRT, and (ii) delivery systems for each of these components. In this review, we will introduce and discuss the various advances in techniques for genetically manipulating murine and human primary T cells mainly in terms of these critical variables. These methods will be assessed in terms of genetic editing of single genes in T cells as well as systematic unbiased studies using genome-scale screening approaches to shed light on novel cellular processes involved in T cell signalling, proliferation, and response to cancer cells. Furthermore, we will discuss the therapeutic application of these genetic editing approaches specifically in the field of T cell-based immunotherapies and CRISPR-based treatments in HIV-AIDS.

## 2. Techniques for Single and Multiplexed Gene Manipulation of T Cells

Careful optimisation of the editing protocol can render most primary T cell subsets amenable to Cas-based genetic manipulation. However, it is important to note that editing efficiency is not the only measure of a successful editing experiment and criteria such as off-target activity as well as post-editing proliferation and cell viability need to be considered. One of the crucial factors influencing the editing efficiency in T cells is their activation state. Stimulated T cells are more susceptible to CRISPR-mediated editing, mostly due to their resistance to harsh treatment since the TCR stimulation enhances their proliferative properties and permissiveness to electroporation as well as viral transduction. Conversely, these properties are not manifested by resting T cells, thereby making them a non-trivial target for CRISPR/Cas9 editing (Figure 2) [17,18,19]. A range of techniques developed for editing of stimulated T cells can be applied to resting T cells, albeit resulting in significantly lower editing efficiencies. However, high efficiency editing of resting T cells would be necessary to gain understanding of processes central to T cell biology such as their activation and differentiation. A number of methods have been specifically developed to edit resting T cells derived from both mice and humans. The section below overviews studies that mainly focus on optimising methods to edit single or multiple genes within both resting and stimulated T cells (Figure 3a,b,d,e).

### 2.1. Single Gene Knockouts

For a single gene knockout, the key components that need to be introduced into the cell, in principle, are a single sgRNA complementary to the target gene sequence and a functional Cas endonuclease (Figure 3a). One of the reasons for the slow progress in editing of primary T cells was the initial difficulty of introducing these components into the cells. Lentiviral vectors, which are commonly used to deliver Cas9 into immortalised cell lines, are not optimal for primary T cells due to low transduction rates [15,16]. Hence the majority of the methods discussed here are mainly based on electroporation to introduce the CRISPR components.

Human T cells: A commonly used method to introduce Cas9 and sgRNA into a cell is electroporation of ribonucleoprotein (RNP) complexes composed of Cas9 protein pre-loaded with sgRNA, a technique initially used to edit activated human T cells by Schumann and co-workers [20]. This pioneering attempt resulted in a limited editing efficiency with indels in the targeted gene identified in ~55% of the T cell population. Quantification of off-target activity at the top candidate off-target sites showed that indel rates at these were below the level of significance. This approach of introducing Cas9 into the cells as a protein and the consequent short-lived endonuclease activity may have been the reason for the reduction of unintended mutations relative to delivery methods resulting in constitutive expression of the enzyme. While the transient activity of Cas9 nuclease arising from electroporation of mRNA or protein is thought to significantly reduce the rates of off-target mutations, genetic engineering of human T cells for therapeutic purposes necessitates further increase in precision of editing. As a consequence, a myriad of high precision Cas9 nucleases have been designed, some of which have been tested in T cells. In a study by Ren and co-workers, a wild-type Cas9 and a high-precision variant eSpCas9 were used to perform a single gene knockout by the means of mRNA electroporation [21]. Sequence-based computational analysis of the target and the off-target sites demonstrated that the use of eSpCas9 reduces the off-target activity to below detection levels while significantly increasing the rate of indels at the target site. A disadvantage of eSpCas9 is that multiple reports have shown that eSpCas9 displays a reduced activity when delivered as RNP [22,23]. To address this, HiFi SpCas9 has been engineered specifically for RNP-based delivery; however, this variant did not lead to meaningful improvement in on-target editing efficiency compared to conventional Cas9.

Even as some of the high precision Cas9 variants remain to be tested and more of such engineered enzymes are in the pipeline, one commonly encountered issue with using Cas endonucleases is the cellular toxicity associated with the resultant DSBs [24]. This has prompted knockout strategies reliant on modified variants of Cas9 such as the Cas-based base editors to be developed for stimulated T cells. Base editors have been previously used to generate exonic pmSTOPs in order to prevent translation, however it has been demonstrated that pmSTOPs can be readthrough with frequencies going up to 30% for some genes [25]. Webber and co-workers have pioneered an alternative approach, whereby a BE4 cytosine deaminase delivered as mRNA into stimulated T cells was employed to mutate splice donor (SD) sites [26]. In this study, mutation of SD site of exon 1 was shown to be more effective for a single gene knockout than introduction of pmSTOPs across two out of the three tested loci. In these two instances the proportion of successfully edited cells approached ~80%.

Apart from the development of the high precision Cas9 variants, a number of other factors have also been optimised for better editing efficiencies including the biological half-life of sgRNA, which has an influence on editing efficiencies. The intracellular exonuclease activity is known to reduce the knockout effectiveness by decreasing the stability of sgRNA. Chemical modification of terminal residues on both 3′ and 5′ ends of sgRNAs have been shown to extend the half-life of sgRNA inside the cell and the use of such synthetic sgRNAs have also been demonstrated to increase the indel rates at target loci by ~3-fold when combined with Cas9 protein electroporation for editing of stimulated T cells. More impressively, such sgRNAs were able to increase the indel rates at target loci from below the detection threshold up to 50% when electroporated along with Cas9 mRNA [27]. Use of such chemically synthesised sgRNAs is now a common practice in the field of genomic editing for cells originating from different lineages [23,26,28,29]. Similarly, extension of the sgRNA half-life by sequential delivery of in vitro transcribed sgRNAs has been demonstrated by Ren and co-workers. Their report shows that an additional electroporation step to deliver sgRNAs 24 h following the initial delivery of sgRNA and Cas9 mRNA by the same means can lead to a ~12-fold increase in the editing performance [21].

An alternative method of delivery of Cas9 into the cells is to electroporate plasmids encoding Cas9 rather than the Cas9 mRNA or the RNPs. However, this method of delivery usually yields a lower editing efficiency as shown by Su and co-workers, who introduced both Cas9 and sgRNA as plasmids constructs and were able to identify indels only in ~30% of the target sites on average [30].

These examples illustrate some of the approaches to genetic manipulation of activated T cells; however the protocols for manipulation of resting T cells have required further optimisations. The earliest reported gene knockout using the CRISPR/Cas9 system in resting T cells was performed by Mandal and co-workers, in which individual plasmids encoding Cas9 and a single B2M-sgRNA were electroporated into human CD4^+^ T cells. This strategy had limited success with the efficiency of the knockout approaching ~5%. The editing performance was improved by simultaneous electroporation of T cells with plasmids encoding two distinct B2M-targeting sgRNAs with efficiencies reaching up to 20% [31]. The original approach relied on conventional sgRNAs which are known to have lower stability inside the cells. Hendel and co-workers were able to improve the efficiency of editing by electroporating chemically modified sgRNA along with Cas9 mRNA into resting human T cells to knockout a gene encoding a chemokine receptor, CCR5, with the editing efficiencies reaching up to ~20% with a single sgRNA [27].

Further improvement in the editing efficiencies in resting T cells was reported by Seki and Rutz, who optimised the RNP electroporation procedure. Using the P2 buffer in combination with the EH100 pulse sequence (Lonza nucleofector 4D) to knock out two genes, CXCR4 and CD127, resulted in CXCR4^-^and CD127^-^ populations reaching up to ~75% and ~40% of bulk CD4^+^ T cells, respectively. Further increases in the knockout efficiencies of both of these genes were observed when three rather than one sgRNAs were used to target the respective loci, resulting in a proportion of successfully edited cells approaching ~90%. This study not only outlines one of the most efficient methods for knockout in resting human T lymphocytes to date, but also recapitulates the notion that using multiple sgRNAs per target locus can elevate the knockout performance [16].

Murine T cells: While murine T cells have a range limitations with respect to simulating their human counterparts, they permit in vivo experiments to be conducted. Thus, murine T cells remain an attractive target of genetic editing. Similar to human T cells, stimulated mouse-derived T cells in vitro can also be manipulated using electroporation techniques to introduce the CRISPR components. A demonstration of this was done by Seki and Rutz, who used electroporation of chemically modified crRNA:tracrRNA and Cas9 protein to delete multiple genes using CM137 electroporation program (Lonza nucleofector 4B). Authors have illustrated the capacity of this method to delete *Pdcd1* and *Ctla4* with the populations of CTLA-4^−^ and PD-1^−^ T cells reaching up to ~90%. In both cases T cells were restimulated for 48h prior to flow cytometric analysis. Given the high editing efficiencies achieved using this method, it has become a benchmark for editing of stimulated murine T cells [16].

Similarly to resting human T cells, resting murine T cells have a poor proliferative capacity and die in the absence of stimulation. To date, two strategies for in vitro knockout in unstimulated murine T cells have been developed, both of which are based on RNP complex electroporation. The first strategy involves stimulation of the resting T cells two hours after electroporation and this approach can lead to editing efficiencies of up to ~60% [16]. Despite the reasonable editing performance, the short time period between RNP delivery and stimulation means that the protein encoded by the targeted gene is unlikely to have been degraded by the time the edited T cells are activated. Therefore, this technique is not suitable to study the role of the disrupted gene in T cell activation and differentiation. The other strategy takes advantage of the finding that the immediate stimulation can be effectively replaced by incubation of the T cells with IL-7, which is known to promote T cell survival, before and after RNP nucleofection. This method can result in ~70% reduction in expression of a target protein in resting T cells and the incubation with IL-7 leads to no apparent activation of the T cells [16].

Murine cells provide a platform for editing T cells in vivo and Beil-Wagner and co-workers were first to carry out such a knockout in unstimulated murine T cells [32]. In their study, oocytes were microinjected with DNA constructs encoding *Cd2*-targeting sgRNA and Cas9 under control of a U6 and a T cell-specific *Cd4* promoter, respectively. Perhaps unsurprisingly, the knockout population constituted only ~1% of T cells isolated from the transgenic mice. The limited success of this approach is likely to have been caused by the inability of the knockout population of T cell precursors to undergo thymic selection, and it is possible that targeting a gene that is not involved in immune cell interactions would have resulted in a better efficiency.

A more viable strategy to perform in vivo knockouts is to directly edit lymphoid cells isolated from constitutive Cas9-expressing mice, which circumvents the need to deliver Cas9 into the cells. To this end, Cas9-expressing mice were developed by Platt et al. by insertion of a Cas9-P2A-EGFP expression cassette into the Rosa26 locus, which has been leveraged by LaFleur et al. to devise a chimera-based Cas9-sgRNA delivery system termed chimera immune editing (CHIME) [33,34]. This technique does not involve direct editing of Cas9-expressing T cells per se, but instead T cell progenitors are edited prior to differentiation into T cells. Briefly, hematopoietic stem cells (HSC) are isolated from the bone marrow of a Cas9 transgenic mouse, lentivirally transduced with a relevant sgRNA and transplanted into irradiated mice. Following the reconstitution of the immune system, T cells are isolated from the chimeric animals and analysed. When CHIME was used to ablate Pdcd1, ~80% of T cells gained indels in the target locus and up to ~90% reduction in expression of Pdcd1 was observed following T cell activation. Mouse strains expressing Cas9-P2A-EGFP have been broadly adopted over the past few years and now are commonly used to isolate Cas9 expressing cells for editing of single and multiple genes or even for performing genome-wide screens. Furthermore, novel constitutive Cas9-expressing mouse strains were developed with improved expression of Cas9 [35].

More recently, Nüssing et al. have developed a method which permits modification of T cells isolated from mice that do not express Cas9. This technique involves RNP delivery into the resting T cells followed by transfer into a congenic mouse strain. In their report, P14 T cells bearing a TCR specific for a lymphocytic choriomeningitis virus (LCMV) antigen were electroporated with *Cd90*-targeting RNP and subsequently transferred into acceptor mice. Rather surprisingly, ~80% of P14 T cells isolated from either LCMV-infected or healthy animals, in which antigenic stimulus was present and absent, respectively, were CD90-negative, demonstrating the utility of this technique to edit both activated and resting T cells [36].

### 2.2. Multiplexed Knockouts

Multiplexed editing permits multiple genes to be edited in a single cell upon delivery of an array of sgRNAs and a Cas enzyme, either a nuclease or a base editor (Figure 3b) [26,37,38,39]. Thus, multiplex knockouts enable faster generation of more complex T cell products. To date, multiplexed editing has only been performed in stimulated T cells.

Human T cells: As with single gene knockouts, RNP electroporation is a viable technique for multiplexed knockouts with the key distinction being that multiple species of RNPs are introduced, each associated with an sgRNA targeting a different gene. Electroporation of RNPs composed of in vitro transcribed sgRNAs and Cas9 protein has been used to perform a double knockout (DKO) and a triple knockout (TKO) in human T cells with the proportion of double negative and triple negative T cells reaching up to 25% and 15%, respectively [40]. Ren and co-workers have also carried out a DKO in T cells using electroporation, but they opted to use Cas9 mRNA instead of the Cas9 protein and incorporated a second electroporation step to deliver another dose of sgRNAs 24 h following the first nucleofection [21]. This approach led to a ~2.5-fold improvement in the DKO relative to the study by Liu and co-workers. The potential of Cas9 mRNA in the context of multiplexed knockouts was further explored by changing the mode of delivery of sgRNAs from electroporation to lentiviral transduction [41]. Optimisation of the lentiviral sgRNA vector revealed that the efficiency of such a multiplexed knockout is highly dependent on the identity of the promoters controlling the expression of the sgRNAs. In addition to the well-established U6 promoter, human H1 and 7SK promoters were found to yield the best performance. This insight led to an increase in the DKO and TKO efficiencies up to ~75% and ~40%, respectively.

One of the risks associated with simultaneous induction of DSBs at multiple loci are chromosomal rearrangements that stem from mismatching of sheared DNA ends by the NHEJ repair machinery. While in the aforementioned studies chromosomal translocation were not detected or the appropriate assays were not carried out, it has been reported that multiplexed knockout in T cells may lead to such genomic defects [42,43]. Therefore, performing multiplexed knockout by means of base editors rather than Cas nucleases is an attractive alternative that has been explored using both cytosine and adenine deaminases [26,44].

Multiplexed base editing in human T cells was demonstrated to yield best results, both in terms of off-target effects and editing efficiencies, when performed by electroporation of the base editor-encoding mRNA along with synthetic sgRNAs [26,44]. Webber and co-workers used this mode of delivery as well as electroporation of cytosine base editor (CBE) BE4 RNP to simultaneously knock out TRAC, B2M and PDCD1 by mutating their splice sites. Optimisation of the BE4 mRNA codon usage and its dose led to TKO efficiency of ~90%. In contrast, the proportion of TKO T cells was 20% lower when BE4 was delivered as RNP. The TKO was accompanied by no detectable genomic rearrangements irrespective of the BE4 delivery mode; however when an identical TKO was performed using Cas9 nuclease instead of BE4, multiple chromosomal translocations between each of the target loci were observed.

Since the development of base editors, there have been multiple reports demonstrating that CBEs including BE4, but not adenine base editors (ABE) have a significant propensity to induce off-target DNA deamination [45,46,47]. Recently developed ABE8-20m base editor, which is a new version of the ABE enzyme designed by site-directed evolution, has shown a capacity to carry out multiplexed knockouts in human T cells when delivered as mRNA [44]. While ABE8-20m was able to simultaneously induce mutations at the splice site of three genes with efficiency of more than 98%, the alteration of the TRAC splice site did not result in loss of TCR expression. The discordance between the editing efficiency on protein and DNA level is likely to be a gene-specific phenomenon which might be alleviated by targeting a different splice site within TRAC. Given the significant off-target effects mediated by CBEs, ABEs are a good alternative for DSB-free editing of T cells.

Murine T cells: Efforts to perform a multiplexed knockout in murine T cells have employed two strategies based on Cas9 nuclease. In vitro DKO was achieved using a knockout strategy developed by Kornete and co-workers, whereby a plasmid encoding Cas9 linked to GFP via a self-cleaving peptide and a single sgRNA was electroporated into primary murine T cells. This was followed by sorting of GFP^+^ cells, in this case equivalent to Cas9^+^ cells, and a re-stimulation [48]. Flow cytometric analysis indicated that DKO was successful in 50% of the GFP^+^ T cells.

In addition to single gene knockouts, the editing strategy developed by Nüssing et al. is also applicable to multiplexed knockouts [36]. Here, LCMV-specific P14 T cells were electroporated with two distinct RNPs and transferred into acceptor animals which were subsequently infected with LCMV to stimulate the transferred T cells. Eight days later, ~80% of the transplanted T cells isolated from the infected mice displayed a double negative phenotype.

### 2.3. Single Gene Knockins

Cas-based gene knockins rely on HDR machinery to install exogenous DNA segments or alter endogenous genomic sequences (Figure 3d). The requirement for an HDRT introduces a number of variables that need to be optimised to fit the T cell-specific constraints. Some of these variables include: (i) The type of template, (ii) mode of its delivery and (iii) features of the template including the size of the insert and the flanking homology arms (HA). HDR is constrained to the S/G2/M phases of the cell cycle, but even then it competes with the NHEJ pathway to repair the DSBs. Consequently, an array of strategies have been developed for primary T cells to enhance the former type of repair. These are exemplified by replacement of Cas9 with either Cas12 or Cas9 nickase, enhancing the shuttling of the HDRT to the nucleus or inhibition of the NHEJ machinery. Given the cell cycle requirements, HDR-based knockins cannot be performed in quiescent or naive T cells and thus cells need to be activated prior to editing.

Human T cells: The first demonstration of a knockin in human primary T cells was carried out by means of electroporation of RNPs and a single-stranded DNA (ssDNA) HDRT to insert a restriction site into a target locus. Optimisation followed by sequencing of the target gene demonstrated that lower concentrations of HDRT may increase the number of total HDR events, but at the cost of reduced HDR to NHEJ ratio. This approach was used to knockin a deleterious restriction site and in effect ablate CXCR4 in T cells. As a result a successful insertion in up to ~25% of the analysed sequences was recorded [20].

Due to their size limitations, ssDNA HDRT templates are considered unsuitable for insertions longer than 100 bp and thus viral or double-stranded DNA (dsDNA) HDRTs have been predominantly used for the purpose of larger knockins. Adeno-associated viruses (AAV) pose a low risk of genomic integration and have a packaging capacity of ~4.5 kb [49]. Vakulskas and co-workers used AAV6 transduction to introduce a polycistronic sequence encoding a CD19-specific CAR (CAR19) and a truncated nerve growth factor receptor (tNGFR) flanked by 400 bp HAs into the TRAC locus [23]. Following delivery of HiFi Cas9 RNP and AAV6 HDRT, surface expression of tNGFR acting as a surrogate for CAR19 was measured to show that a successful knockin occurred in ~10% of T cells. A significant enhancement in HDR was observed in a similar study published by Eyquem and co-workers who also used an AAV vector to replace endogenous TCR with CAR19; however instead of using a Cas9 variant RNP, conventional Cas9 was delivered as mRNA [28]. Additionally, the HDRT consisted of an insert lacking the tNGFR expression reporter, flanked by longer 950 bp HAs. These alterations to the editing protocol led to a 5-fold improvement in the knockin efficiency relative to that observed by Vakulskas and co-workers. This is consistent with reports suggesting that longer HAs and shorter inserts are associated with an increase and reduction of the knockin efficiency, respectively [50].

One concern regarding the delivery of dsDNA HDRT by AAV vectors is the presence of AAV-specific immunity in humans, which could lead to poor engraftment of therapeutic T cells manufactured using this method [51,52,53]. Therefore, direct delivery of naked dsDNA would be preferred for engineering of cell products for clinical applications. However, naked dsDNA is associated with cellular toxicity. In an effort to resolve this issue, a revised protocol for electroporation-based delivery of dsDNA HDRT and Cas9 RNPs has been developed [29]. In brief, HDRT and RNPs were pre-mixed, briefly incubated, resuspended with T cells and electroporated using an EH115 pulse sequence (Lonza nucleofector 4D). Optimising the concentration of the dsDNA HDRT was critical to ensure reasonable cell viability without significantly compromising the knockin efficiency. Similarly, the order in which the cells, RNP and HDRT were combined had a considerable impact on the cell survival: mixing of RNP with HDRT prior to addition of cells and electroporation could increase the editing efficiency by ~3-fold albeit at the cost of reduced T cell viability. The capacity of this method was tested in an attempt to insert a polycistronic sequence encoding α and β chains of a transgenic TCR into the endogenous TRAC locus. Integration of an HDRT template composed of a 1.5 kb insert and 300 bp HAs into a target was successful in ~10% of edited T cells.

Given the success of dsDNA HDRT to mediate insertion of large segments, its potential to replace ssDNA HDRT as a template for short insertions and point mutations has also been tested. In order to repair deleterious point mutations in an interleukin receptor encoding gene, Roth and co-workers designed 600 bp long dsDNA and 120 bp ssDNA HDRTs [29]. Compared to ssDNA HDRT, the use dsDNA HDRT was able to restore expression of this receptor in 30% more T cells across two donors.

These studies illustrate that there have been significant advancements in methods for generating knockins in T cells. Nonetheless, the achievable knockin efficiencies mean that the successfully engineered cell population needs to be enriched following the editing procedure. While the enrichment may be straightforward for transgenes expressed on the cell surface, selection of cells bearing edits in genes encoding intracellular proteins may pose technical challenges, especially when using flow cytometry-based technology that requires membrane permeabilisation prior to antibody staining.

Recently, there has been an outpouring of techniques aimed at improving efficiency of HDR-based editing in T cells. Cas12a is another member of the CRISPR/Cas nuclease family whose activity in the presence of HDRT appears to display a stronger preference for HDR over NHEJ than Cas9 [54]. This might be a consequence of the ability of Cas12a to induce DSB outside the target sequence which permits for re-cleavage following failed attempts to repair the DSBs by the HDR rather than the NHEJ machinery [54,55]. This property of Cas12a can be leveraged to effectively increase the knockin efficiency. Delivery of Cas12a mRNA and AAV6 carrying the HDRT as well as crRNA into T cells led to a successful installation of a ~1 kb insert with ~45% efficiency on both DNA and protein level [38].

An alternative approach to increase HDR is to enhance shuttling of the HDRT to the nucleus where the editing occurs. While nuclear import of Cas9 can be ensured by addition of one or more nuclear localisation signals (NLS) to its terminus, promoting nuclear localisation of HDRT is more challenging. Nguyen and co-workers have shown that knockin efficiency can be improved by appending dsDNA HDRTs with short DNA sequences, which can be bound but not cleaved by Cas9 [56]. In consequence, Cas9 RNPs which contain the NLS are enlisted to act as a nuclear shuttle for the tagged dsDNA template. This strategy has shown a tremendous ability to enhance knockin of large DNA segments into a range of loci in T cells; in most extreme cases, addition of the tag to the dsDNA template could increase the HDR efficiency by ~4-fold. Another method that enhances the delivery of the HDRT has been developed by Gwiazda and co-workers. This technique relies on co-electroporation of mRNA encoding Cas9 and adenoviral proteins which increase the permissiveness of primary T cells to AAV transduction. As such, the utility of this approach is restricted to HDRTs delivered by AAV vectors [57]. Nonetheless, this method permits HDR efficiencies to be improved by up to ~2-fold.

In addition to modifying the substrates to favour HDR, the components of the repair pathways as well as their regulatory networks can be directly modulated to diminish the probability of NHEJ. Several small molecules have been shown to enhance HDR, but up to now only a few of them have been validated in T cells [58,59,60]. XL314 is an inhibitor of a cell cycle kinase CDC7 which was shown to enhance HDR in primary T cells, possibly by extending the time that the cell remains in the HDR-permissive phases [61]. Treatment of T cells with XL314 following Cas9 RNP delivery was able to increase the total knockin rate using ssDNA and dsDNA HDRTs by ~2-fold and ~1.2-fold, respectively. Moreover, its use increases the HDR to NHEJ ratio as well as the proportion of cells with biallelic knockins.

Murine T cells: The methods designed for in vitro knockins in murine T cells are similar to those for human T cells. As with human T cells, the length of HAs and the insert size are crucial variables for achieving higher efficiency as shown in two studies [36,48]. Nonetheless, neither of these studies demonstrates knockin rates comparable to those previously described for human T cells. Systematic testing of the approaches developed in human T cells or development of mouse-specific strategies for HDR enhancement will be the key to improving the knockin efficiencies in murine T cells.

### 2.4. Multiplexed Gene Knockins

Engineering of more complex cell products, especially for therapeutic purposes may require introduction of multiple genes into a single T cell (Figure 3e). Ability to do so in a single step is more likely to yield a uniform population of T cells expressing each of the transgenes. However, the compounding of the individual knockin efficiencies and intrinsic capacity of cells to absorb a limited amount of the editing agent mean that multiplexed knockins are technically challenging. Here, we will discuss the recent methods that have addressed these issues.

Human T cells: Early attempts to perform a double knockin (DKI) in primary T cells were carried out by Dai and co-workers using Cas9 mRNA and AAV6 carrying sgRNAs and HDRTs, albeit with a very low efficiency of ~3% [38]. The authors however were able to substantially improve the editing efficiency by using Cas12a instead of the conventional Cas9. Apart from Cas12a favouring HDR over NHEJ, it also has the advantage that it only requires crRNA for full functionality and its RNAse activity permits processing of multiple crRNAs from an RNA array, thus a single promoter is sufficient to drive expression of multiple crRNAs from a viral vector [55]. The authors used AAV-based HDRT templates along with Cas12a mRNA to generate fusion proteins as well as introduce large genes. In the latter case, knockin of genes encoding two CARs into two distinct loci was achieved with a ~35% efficiency.

Further improvement in multiplexed editing was achieved by leveraging the method based on electroporation of Cas9 RNPs and dsDNA HDRT which was developed by Roth and co-workers [29]. Double knockins (DKI) and triple knockins (TKI) using dsDNA HDRTs containing 300 bp HAs have led to editing efficiencies of up to 20% and 1.5%, respectively. However, the DKI efficiency has varied considerably between different sets of targeted loci and inserted sequences, thereby highlighting the need for more robust techniques to be developed for Cas-based multiplexed knockins.

Murine T cells: To date, there has been a single report describing a multiplexed knockin in murine T cells. In order to induce point mutations in two distinct genes, plasmids encoding Cas9, sgRNAs and the HDRTs were electroporated into T cells. Disappointingly, the proportion of T cells expressing the edited genes was ~2%. While the authors did not elaborate on the possible cause of the low efficiency of the DKI, it is not unlikely that better performance could be achieved by delivering the endonuclease in the form of mRNA or protein [48].

## 3. Techniques for Pooled Genetic Manipulation

### 3.1. Pooled Knockout Screens

Pooled knockout screens permit a systematic identification of genes engaged in a wide range of cellular processes (Figure 3c). Due to a well-documented involvement of T cells in autoimmune conditions and cancer immunobiology, there has been a growing interest in the regulatory networks that underlie their differentiation, proliferation and effector functions. Initial attempts to carry out pooled genetic screens in T cells relied on RNA interference (RNAi) technology [62,63]. However, the improved precision of the Cas enzymes led to RNAi being largely superseded by CRISPR/Cas9. Nonetheless, there is a set of challenges associated with CRISPR/Cas9-based screens in T cells such as the requirement for uniform expression of the Cas9 enzyme in the cell population and efficient delivery of sgRNAs. Despite a growing number of methods to accommodate various T cell phenotypes, each CRISPR/Cas9 screen follows a set framework. In short, sgRNAs and Cas9 are delivered to a cell population to create a mutant library of cells and positive, negative or marker-based selection screens are performed. Negative selection screens are carried out in order to identify the genes that are essential for the survival and proliferation of the cells. In such screens, cells are mutagenised and the mutant population is cultured over a period of time. Cells that harbor mutations in genes that are essential for proliferation will eventually die during this period of time leading to ‘drop-out’ of the cells from the population. By comparing the abundance of the sgRNA present at the endpoint of the experiment to the original population, the genes that are essential for the process, i.e., in this case the genes whose guides are under-represented, can then be inferred. In a positive selection screen, a selective pressure (e.g., drug) is added to the cells and mutant cells that are enriched are collected to identify the genes that are responsible for the observed phenotype. In a marker-based screen, the desired phenotype is usually an expression- or a fluorescence-marker or a drug resistance cassette, based on which selections can be made. In all cases, the identity of sgRNAs targeting specific genes that are enriched in the cell population of interest, referred to as hits, is established by deep sequencing followed by analysis of the relative sgRNA enrichment between the target and control cell populations [64]. It is worth noting that the composition of the sgRNA libraries can be restricted to refine the screen to a set of predetermined genes. Thus, a pooled knockout screen can be designed to interrogate the function of a subset of genes involved in a function of interest as well as all protein-coding genes—often referred to as a genome-wide screen. In this section, we will examine the techniques developed for pooled knockouts in different subsets of T cells and discuss the recent emergence of knockin screens.

Human T cells: Lentiviral transduction is an efficient method for delivery of sgRNA libraries into primary T cells. This is not the case for Cas9 whose delivery via lentiviral transduction is associated with poor expression and thus it is delivered by electroporation of recombinant protein. The combination of these two methods, referred to as sgRNA lentiviral infection with Cas9 protein electroporation (SLICE), has been used to perform genome-wide screens coupled to immunological and genetic assays. For instance, SLICE was combined with proliferation assays to identify genes involved in CD8^+^ T cell activation [65]. This screen has been able to effectively identify well-known positive and negative regulators of cell activation. SLICE has also been incorporated into a CROP-seq workflow, whereby following a pooled knockout, CD8^+^ T cells can be subjected to scRNA-seq [66,67]. As a result, loss-of-function mutations can be linked to a transcriptomic profile associated with a cell state of interest.

Despite the success of SLICE in CD8^+^ T cells, Ting and co-workers demonstrated that in CD4^+^ T cells SLICE may result in inconsistent knockout efficiency between different sgRNAs targeting the same gene [68]. The authors demonstrated that electroporation of CD4^+^ T cells with Cas9 RNPs bound to non-targeting sgRNAs, rather than recombinant Cas9 following lentiviral delivery of targeting sgRNAs, can improve the knockout efficiency and in effect reduce the inter-sgRNA variation. This feature is fundamental for accurate identification of hits since the sgRNA enrichment analysis software relies on ranking of multiple sgRNAs targeting the same gene. This method, known as Guide Swap has been effectively used to perform a genome-wide screen for genes regulating expression of three well-characterised T cell surface proteins; however, direct comparison of SLICE and Guide Swap in the same T cell subset has not been performed.

Murine T cells: The advent of Cas9 transgenic mouse strains permitted CRISPR/Cas9-based pooled knockout screens to be performed in vitro as well as in vivo [33,34]. A major advantage of using T cells derived from Cas9 expressing transgenic mice is the complete redundancy of the Cas9 delivery step, which is often the bottleneck in performing genome editing at scale. Furthermore, in vivo knockout screens permit dissection of more complex processes such as anti-cancer immunity which inherently involve complicated cell-cell interactions and thus are not easily simulated ex vivo. Despite the limitations of the mouse models with respect to the human immune system, CRISPR/Cas9 screens in mice have led to relevant findings that could be validated in human T cells.

Similarly to human T cells, murine CD8^+^ T cells are permissive to lentiviral delivery of sgRNA libraries. Dong and co-workers set out to perform a genome-wide screen to determine genes contributing to infiltration of tumours by T cells [69]. To this end, OT1 Cas9 CD8^+^ T cells were transduced with a genome-wide sgRNA lentiviral library and subsequently transplanted into immunodeficient mice bearing OVA-expressing tumours. To analyse the sgRNA enrichment, CD8^+^ T cells were isolated from the population of tumor-infiltrating lymphocytes (TIL). Comparative analysis of sgRNA enrichment in T cells transduced with the genome-wide library relative to a control population led to identification of genes previously not known to be involved in regulating tumor infiltration. An analogous screen was also performed to investigate the genes contributing to T cell cytotoxicity; however, instead of transferring the T cells into mice, T cells were incubated with the cancer cells in vitro. Finally, a T cell population displaying high density of a degranulation marker was isolated and analysed.

Unlike lentiviruses, AAVs rarely integrate into the host genome and thus are of limited use for delivery of sgRNA libraries since the sequence of an sgRNA cannot be recovered by standard sequencing techniques. Nonetheless, AAVs have been readapted for the purpose of pooled knockout screens in the form of an AAV-Sleeping Beauty (SB) Transposon hybrid system [70]. Upon infection with the AAV-SB hybrid virus, a strong promoter drives the expression of an SB100X transposase which subsequently integrates the AAV-delivered insert containing the sgRNA into the host genome. Advantageously, SB100X-mediated integration displays a preference for “safe haven” regions of the genome and permits the sgRNA identification by high-throughput sequencing methods. The AAV-SB system has been used in the context of in vivo CRISPR knockout screens to identify a repertoire of immunotherapy targets for treatment of immunologically ‘cold’ tumours. Briefly, CD8^+^ T cells were transduced with the AAV-SB-based library targeting genes encoding cell surface proteins, transferred into mice bearing glioblastoma tumours, at the endpoint TILs were recovered and sequenced.

Murine CD4^+^ T cells are only weakly susceptible to lentiviral infections and in order to carry out CRISPR/Cas9 screens in this T cell subset retroviral sgRNA libraries have been developed. A pioneering retrovirus-based screen was performed in naive CD4^+^ T cells isolated from Cas9 mice to identify genes involved in regulation of CD4^+^ T cell differentiation [71]. Unlike in the lentiviral screens, in this instance T cells were subjected to an antibiotic selection following the delivery of the sgRNA library to increase the proportion of sgRNA-expressing T cells. Retroviral delivery has also been found to be applicable to pooled screens in regulatory T cells. In a study by Cortez and co-workers, an sgRNA library restricted to genes encoding nuclear proteins was used to identify regulators of a master transcription factor FoxP3 [72]. Here, delivery of the sgRNA library was enhanced by an additional round of retroviral transduction which obviated the need for antibiotic treatment. The success of retroviral libraries to dissect the functions of murine CD4^+^ T cells sets a template for future use of pooled knockouts in this cell type.

The aforementioned CHIME workflow has also been used for in vivo pooled knockout screens [34]. HSCs were extracted from constitutive Cas9-expressing mice, transduced with an sgRNA library and transferred into congenic animals. Following immune reconstitution, CD8^+^ T cells expressing a fluorescent transduction marker were isolated and injected into recipient mice where T cells can be subjected to a selection pressure such as a viral infection. Eight days after selection the edited T cells can be isolated from the recipient mice by means of cell sorting for congenic markers.

### 3.2. Pooled Knockin Screens

Thus far, the pooled CRISPR/Cas9 screens have been limited to identification of endogenous genes contributing to various cellular processes and did not have means to evaluate the influence of exogenous constructs on T cell functions. To this end, a method for pooled knockin screens was developed which combines simultaneous delivery of a library of dsDNA HDRTs and Cas9 RNPs by electroporation (Figure 3f) [73]. The HDRTs are integrated into a single locus specified by the sgRNA to generate a T cell population expressing on average one insert per cell. The delivery of the library is followed by assays to evaluate the impact of each construct on T cells; however, rather than analysing the endogenous gene depletion as in the case of the pooled knock-out screens, enrichment of the inserts is measured instead. It was demonstrated that the pooled knockin screens can be coupled to proliferation assays as well as scRNA-seq, both in vitro and in vivo. Together, these methods and the pooled knockin have been applied to screen heterologous immunoreceptors and transcription factors for their capacity to improve anti-cancer activity of human T cells.

## 4. Clinical Applications of Edited Primary T Cells

### 4.1. Editing of T Cells for Cancer Immunotherapy

Among the many important immune functions, the role T cells play in cancer progression is one of most extensively studied. In the early stages of development of tumour cells, cytotoxic CD8^+^ T cells have the potential to recognize and eliminate immunogenic cancer cells. Cancer cells however can avoid clearance mediated by T cells mainly by impairing their effector functions and proliferative capacity [74]. This is often achieved by a number of mechanisms; one such mechanism involves the engagement of inhibitory receptors (IR) of T cells to downregulate their activity. For example, some cancer cell types express PD-L1 on their surface, which is a ligand for the negative regulator of T cell function PD-1. The engagement of PD-1 with PD-L1 leads to inhibition of T cell signalling thereby effectively decreasing anti-tumor activities such as T cell migration, proliferation and secretion of cytotoxic granules. The past few years has seen a number of therapeutics being developed to counteract this immune evasion mechanism. Immune checkpoint blockade (ICB) of mainly PD-1 and CTLA-4 has been shown to increase the immune response of cytotoxic T cells towards different types of solid tumours. Nonetheless, there is a range of malignancies that remain unresponsive to this line of immunotherapy and efforts are being continuously made to improve immunotherapy approaches [5].

The ability to genetically manipulate T cells has led to a revolution in the field of oncoimmunology, hallmarked by the approval of T cell-based therapeutics for treatment of B cell malignancies. T cells engineered using early gene editing technologies such as TALENs or ZFNs made headway in the direction of T cells with enhanced therapeutic potential, but the ease of reprogrammability offered by Cas-based tools has significantly advanced the field. In this section, we will briefly review the specific applications of Cas enzymes in the context of therapeutic T cells. For a more detailed discussion of gene editing in the context of immunotherapies readers are directed to other excellent reviews [75,76,77].

Delivery of TAA-specific receptors: The cytolytic capacity of T cells can be redirected against malignancies by endowing them with a receptor specific to a relevant TAA. This receptor can either be a TCR or a CAR with the key distinction between the two being their ability to target different types of antigens—the former can recognise cytoplasmic neoantigens, whereas the latter is restricted to cell surface proteins. Treatments relying on infusion of patients with either physiological or gene-edited T cells is referred to as adoptive cell therapy (ACT); here this term will be used in reference to CAR and TCR-transgenic T cells.

Traditionally, TAA-specific TCRs and CARs have been introduced into autologous T cells by retroviral or lentiviral transduction. The semi-random integration of the viral vectors may lead to oncogenic transformation or a heterogeneous expression of the receptor across the engineered T cell population. Furthermore, the expression of the endogenous TCR is downregulated following antigenic stimulation—this safeguard against a detrimental immune response is not conferred by the viral delivery of the TAA-specific receptor [78]. Additionally, the overexpression of the virally delivered CAR has been demonstrated to lead to T cell exhaustion stemming from tonic signalling. These issues have been addressed by HDR-mediated integration of constructs encoding TAA-specific receptors into *TRAC* or *TRBC*. To do so, either of the two TCR loci can be targeted by Cas delivered as mRNA or RNP [23,28,29,38]. Effective delivery of HDRTs encoding a TAA-specific receptor was demonstrated using AAV transduction as well as dsDNA electroporation. However, given the evidence suggesting pre-existing immunity to AAVs in humans, the latter mode of HDRT delivery may prove to be safer in clinical settings.

It must be noted that conditional on the HDRT design and the position of the sgRNA target sequence within the locus, HDR-mediated insertion of the TAA-specific receptors can be engineered to lead to a concurrent disruption of the gene at the target locus. Thus, the knockin can be handily coupled to knockout of other genes whose deletion may lead to improved clinical efficacy of ACT.

Beyond enabling physiological regulation of expression of the TAA-specific receptor, Cas-based editing has been used to resolve the issue of TCR chain mispairing. Following viral delivery, chains of the transgenic and endogenous TCR can form hybrid receptors composed of a transgenic TCRα and endogenous TCRβ and vice versa. Such mixed TCR dimers have unpredictable, potentially autoreactive specificities which can lead to graft versus host disease (GvHD). Additionally, the presence of the endogenous TCR reduces the surface expression of the transgenic TCR and in consequence curtails antigen sensitivity of the engineered T cell. While a knockout of a single endogenous TCR chain can partially restore the sensitivity of TCR-transgenic T cells, it does not prevent formation of the mixed TCR dimers [79,80]. Therefore, disruption of both *TRAC* and *TRBC* loci in TCR-transgenic T cells is highly desirable. To this end, dsDNA HDRT and Cas9 RNP electroporation has been effectively used to knockin a polycistronic construct encoding both transgenic TCR chains into one of the TCR loci and perform a knockout at the other loci [29,80]. An alternative editing strategy, albeit less efficient, relies on a multiplexed knockin of individual transgenic TCR chains into the two endogenous TCR loci [29,80]. Both approaches have been demonstrated to eliminate the chain mispairing as well as confer native-like control of transgene expression.

Recently, a first clinical trial of CRISPR-edited TCR-transgenic T cells was completed [81]. In this study, *TRAC* and *TRBC* were deleted by means of Cas9 RNP electroporation prior to lentiviral transduction of a transgenic TCR to preclude the formation of mixed TCR dimers. Consistently, no adverse effects associated with GvHD were reported. Given the evidence for widespread immunity to Cas9 [82], the authors have further shown that neither of the patients mounted an immune response towards the protein following the T cell infusion. These findings are likely to further reinforce the status of RNP electroporation as a preferred mode of Cas9 delivery for manufacturing of therapeutic cell products.

CARs employed in both of the clinically approved T cell therapies recognise a B cell antigen known as CD19. Despite their success in the clinic, targeting a single TAA is associated with a risk of antigen escape and relapse. To ensure long-term remission, CAR T cells expressing two distinct TAA-specific receptors have been engineered to target antigens co-expressed on the surface of neoplastic cells. Manufacturing of such dual CAR T cells with the CAR transgenes integrated into the TCR loci can be streamlined by means of multiplexed knockins [29,80].

Ablation of immune checkpoints: Exposure of T cells to the immunosuppressive tumour microenvironment (TME) is associated with T cell dysfunction characterised by impaired effector functions, persistence and failure to infiltrate the tumour. ICB serves to reinvigorate such exhausted T cells and has led to unprecedented clinical outcomes in patients with solid tumours. In contrast, the clinical success of ACT has been largely limited to blood cell malignancies.

While clinical trials combining ACT and ICB are underway, systemic administration of ICB antibodies can lead to adverse effects associated with an overactivated immune system [83]. To circumvent the side effects, ICB can be applied in a more localised manner by deletion of IRs in the engineered T cells. Mirroring the antibody-mediated ICB, inhibition of the PD1-PDL1 signalling axis was predominantly tested in preclinical and clinical trials of ACT-based ICB. Ablation of PD-1 in CAR T cells using either Cas9 RNP or mRNA electroporation has been demonstrated to enhance the clearance of PD-L1 expressing tumours in mice [21,84]. Furthermore, the safety of PD-1 deficient TCR-transgenic T cells was demonstrated in a clinical setting [81].

Augmentation of intracellular signalling: Even though ICB has proved to be an effective therapy for a small group of cancers, there is a significant subset of patients whose tumours remain refractory to this line of treatment. This could be aided by identification of novel targets for antibody-based immunotherapy, however only a fraction of the proteome is located on the T cell surface and thus is accessible to antibodies. By contrast, genetic editing of T cells provides access to a wide spectrum of intracellular signalling components whose disruption could potentially improve anti-tumour activity of T cells. Identification of novel cytoplasmic targets has been aided by in vivo and in vitro pooled knockout screens with a number of candidates validated in tumour xenograft mouse model [69,70]. For instance, Wei and co-workers found that a deletion of an RNase called REGNASE-1 can augment the capacity of cancer-specific T cells to control tumour growth by enhancing their ability to infiltrate and persist within the malignant tissue [85]. Similarly, a knockout of a transcription factor GATA-3 leads to improvements in the cytolytic properties of T cells [86]. Evidence showing that simultaneous knockout of multiple intracellular targets synergistically enhances the anti-tumour potential of ACT highlights the need for development of techniques for more robust multiplexed editing [85].

Reduction of fratricide: The progress in development of ACT for T cell malignancies has been stymied by lack of appropriate TAAs. Since antigens such as CD5 and CD7 are ubiquitously expressed on the surface of healthy as well as neoplastic T cells [87,88,89], CAR T cells targeting these antigens display fratricidal activity affecting their therapeutic potential [90,91,92]. To circumvent these limitations, Cas9-mediated disruption of these TAAs has been tested. For instance, ablation of CD7 in autologous CAR T cells targeting this surface receptor significantly reduced the fratricide and improved survival of tumour xenograft mice treated with such T cell products [90,91].

Universal allogeneic CAR and TCR-transgenic T cells: The manufacturing process of ACT is logistically challenging and requires substantial resources that hinder widespread access to the treatment. Furthermore, patients displaying low white blood count may not be able to donate a sufficient number of T cells for production of a T cell-based therapeutic. These challenges could be resolved by engineering of universal allogeneic ACT which would obviate the need for use of autologous T cells [93]. Production of such ‘off-the-shelf’ cell products requires the cell surface expression of the HLA, which is the human version of the MHCI, and endogenous TCR to be disrupted. Knockout of the former molecule is necessary since the patient’s immune system may mount a response against the allogeneic HLA leading to rejection of the engineered T cells, whereas the endogenous TCR needs to be ablated because it may recognise alloantigens and induce the GvHD [94,95,96]. While both TCR and HLA form complexes composed of multiple polypeptide chains, knockout of a single constituent is sufficient to abolish surface expression of either protein [97]. Due to the polyallelic nature of the heavy chain of HLA*, B2M* is a preferable knockout target whose disruption abolishes surface expression of all *HLA* alleles [97].

### 4.2. Editing of T Cells for Antiretroviral Therapies

HIV is a blood-borne virus which primarily infects CD4^+^ T cells. To gain entry into the cell, viral envelope glycoproteins bind to the CD4 molecule and either CCR5 or CXCR4, depending on the tropism of the HIV strain. Subsequently, the HIV single-stranded RNA genome is retrotranscribed to dsDNA and integrated into the host genome where it can either be actively expressed, killing the host cell in the process or can adopt a latent state with a potential for reactivation. The latent reservoir is predominantly located in CD4^+^ memory T cells, but it has also been found in other tissues. Treatment of HIV patients using a cocktail of small molecule inhibitors termed highly-active antiretroviral therapy (HAART) can effectively suppress viral replication allowing the acquired immunodeficiency syndrome to be averted. Nonetheless, the treatment is not curative since it does not lead to eradication of the latent reservoir which gradually reactivates following interruption of HAART. However, elimination of the latent reservoir could be achieved by in vivo delivery of gene editing tools to induce inactivating indels in the HIV genes or excision of the provirus by cutting its flanking sequences. Removal of the provirus from the latently infected cells is not the sole path to achieving long-term remission. It has been postulated that editing of autologous CD4^+^ T cells to disrupt the viral entry co-receptors followed by infusion of the CCR5- or CXCR4-deficient T cells into the patients could lead to elimination of the reservoir by repopulating the lymphoid system with HIV-resistant cells. Here, we will summarise how the CRISPR/Cas9 editing technology has led to progress in the development of a curative HIV therapy and the associated technical challenges. We direct readers seeking a more comprehensive discussion of the field towards the following reviews [98,99].

Eradication of the latent HIV reservoir: The HIV ~10 kb provirus is flanked by long terminal repeats (LTRs) whose sequences are identical at the time of integration, but diverge in the course of infection. Nonetheless, the high degree of sequence identity between the 5′ and the 3′ LTRs permits excision of the provirus using a single sgRNA, thus making the LTRs a convenient target for Cas-based editing. The feasibility of LTR targeting by Cas9 to remove the provirus ex vivo has been first demonstrated in Jurkat T cell lines modified to act as surrogates of latent infection [100,101]. Replicating this editing strategy, Kaminski and co-workers used lentiviral vectors to deliver the CRISPR components to primary CD4^+^ T cells obtained from HIV-positive patients [102]. As a result of this treatment, the copy number of the provirus was reduced by up to 90%. In addition to employing Cas9 to cleave the provirus, it can be also be efficiently used to degrade the intermediate HIV dsDNA genome prior to its integration and establishment of the viral reservoir [103]. While this strategy can lead to effective protection of primary CD4^+^ T cells ex vivo, it has a limited potential in the clinic since it requires constitutive expression of the Cas9 endonuclease. As a consequence, the preclinical in vivo studies have largely focused on excision of the provirus by the transient introduction of Cas9 and sgRNAs into latently infected cells.

The key challenge of in vivo elimination of the viral reservoir is the systemic administration of Cas9 to the T cells containing the provirus. To this end, injections of model animals with CRISPR-encoding AAV vectors have been used to deliver the endonuclease and sgRNAs. The limited packaging capacity of AAVs necessitates the use of smaller Cas9 orthologs such as SaCas9 rather than the more widely adopted SpCas9 [49]. To improve the efficacy of the treatment a second sgRNA targeting the viral gene Gag is typically used in the in vivo setting in addition to the LTR-specific sgRNAs. Such CRISPR-based therapy needs to be stringently tested for any off-target activity of Cas9.

In a proof-of-concept study, AAV9-packaged Cas9 and sgRNA were deployed to eliminate a replication incompetent provirus in a mouse model [104]. This strategy was also tested in an improved model of human HIV infection, namely humanised mice inoculated with a replicating HIV strain [105]. While both studies demonstrated that AAV-delivered CRISPR is capable of reducing the viral reservoir in a range of tissues, neither reported a complete eradication of the latent infection. More recently, Dash and co-workers combined AAV9-packaged CRISPR with a novel type of antiretroviral therapy to control the HIV infection and eliminate the viral reservoir in humanised mice [106]. Such treatment has been successful in a number of animals with no Cas9-associated off-target effects detected by whole-genome sequencing.

Disruption of the viral entry co-receptors: Entry of the virus into a cell requires the envelope glycoprotein of HIV to sequentially engage CD4 and one of the co-receptors—either CCR5 or CXCR4. While ablation of *CD4* expression would confer HIV-resistance onto the edited T cells, CD4 is an essential surface receptor for immune function and development. Editing of the co-receptor genes, *CCR5* in particular, is an alternative pathway to generate HIV-resistant autologous T cells which can be transplanted into HIV-positive patients. This strategy to achieve a long-term remission of the virus was pursued ever since an HIV-positive leukaemia patient was effectively cured by a bone marrow transplant from a *CCR5Δ32/Δ32* donor [107]. The homozygous 32 bp deletion in *CCR5* is present in the Caucasian population at low frequency and is associated with a complete loss of the co-receptor surface expression, thereby showing that *CCR5* can be safely ablated without impairing the function of T cells. The first genetically modified CCR5-deficient T cells that were transplanted into patients were edited using the ZFN technology [108]. While the patients enrolled in the trial did not experience any side effects related to potential off-target activity of the editing agent, CRISPR/Cas9 has a superior precision and thus is better suited for production of edited autologous cell products [108]. Consequently, there are multiple studies reporting Cas9-based editing of both co-receptors, individually and simultaneously, in primary CD4^+^ T cells.

Cas9 has been successfully used ex vivo in primary CD4^+^ T cells to disrupt the expression of *CCR5* by the introduction of indels at the sgRNA target site as well as introduction of the *CCR5Δ32* allele by excision of a relevant gene fragment [109,110]. These edits were achieved using CRISPR components delivered by adenoviral and lentiviral vectors, respectively. Similarly, *CXCR4* was ablated in primary CD4^+^ T cells by plasmid-deployed SpCas9 [111]. While it is widely accepted that transduction and transfection are inadequate modes of Cas9 delivery due to the resulting low editing efficiency, the co-receptor-deficient T cells generated in the aforementioned studies have displayed a reduced susceptibility to the virus. However, ablation of a single co-receptor may not confer HIV resistance onto the in vivo edited T cells since HIV is capable of shifting its tropism from CCR5 to CXCR4 over the course of infection [112]. To address this, Cas9 has been used to simultaneously knockout both co-receptors in primary CD4^+^ T cells with the resulting cell populations showing protection against CCR5- and CXCR4-tropic HIV strains [113,114].

With the prospect of *CXCR4* and *CCR5* editing in vivo, several smaller Cas variants that can be more efficiently packaged into AAV vectors have been tested. SaCas9 has been used to knock out *CXCR4*, whereas more recently *CCR5* was ablated by Cas12a [115,116]. In vivo knockout of the co-receptors would permit a significant reduction in the cost of such treatment if it is approved for clinical use in the future.

Infusion of patients with CCR5-deficient autologous T cells is unlikely to lead to side effects as *CCR5Δ32/Δ32* individuals do not suffer from any major pathophysiologies. By contrast, multiple functions of CXCR4 in the human immune system have been reported and C*xcr4*-deficient mice die early in development. Thus, site-specific manipulation of *CXCR4* that blocks viral entry, but not its interactions with the physiological ligand is desirable. To this end, Tian and co-workers identified mutations in *CXCR4* that selectively obstruct binding of the HIV envelope glycoprotein [117]. Delivery of CRISPR components along with appropriate HDRTs into primary CD4^+^ T cells should yield *CXCR4*-mutant cells that could be safely transferred into patients. HDR-based manipulation of *CXCR4* has been previously demonstrated in CD4^+^ T cells, albeit not with the purpose of inserting the HIV-blocking mutations [19]. Ideally, the knockin of the HIV-blocking point mutation into *CXCR4* would be combined with a knockout of the other co-receptor; editing strategies such as the one developed by Dai and co-workers could enable the multiplexed edit with high efficiency and speed [38].

## 5. Summary and Future Directions

As is evident from many studies discussed here electroporation has become the method of choice for Cas9 delivery for in vitro knockouts in both murine and human primary T cells. While the procedure has become very well optimised and further improvements are continuously being made, its effects on T cell function have been poorly characterised. In a recent study, analysis of gene expression profiles of resting T cells following an electroporation demonstrated a dramatic dysregulation of genes involved in T cell activation [118]. An alternative Cas9 delivery method termed microfluidic squeezing markedly reduces the dysregulation of the immune signalling pathways without the loss of efficiency [118]. Notwithstanding these findings, microfluidic squeezing has not been widely adopted perhaps due to limited commercial availability of the microfluidic devices.

The advancements in the field of genome editing have been very useful for improving the techniques available for editing of primary T cells. As we have discussed, apart from the conventional Cas9, many strategies for effective editing of primary T cells are reliant on CBEs or ABEs. The newly described prime editor, which is a catalytically impaired Cas9 endonuclease fused to an engineered reverse transcriptase, has been shown to be capable of directly writing new genetic information into a specified locus without creating DSBs or having the limitations of CBEs or ABEs of being able to only convert C to T and G to A, respectively [119]. Prime editors are yet to be used for editing of primary T cells and currently the utility of prime editors is thought to be restricted by delivery options, as these enzymes tend to be much larger than conventional Cas9. However, as delivery techniques improve, this would be a very powerful tool for creating precise edits for primary T cells, which would be highly desired for therapeutic applications.

Currently, engineering of TCR-transgenic and CAR T cells predominantly relies on retroviral or lentiviral delivery of the constructs encoding the TAA-specific receptors. However, targeting of the receptor-encoding constructs to endogenous TCR loci confers multiple advantages which could ultimately translate into better clinical outcomes and reduced adverse effects of ACT. We discussed a number of CRISPR-based techniques that have emerged, which permit a knockin of large exogenous sequences with high precision in primary T cells [28,29,38]. Findings showing that TCR-transgenic T cells manufactured using the CRISPR/Cas9 system do not induce Cas-specific immune responses will permit these techniques to be more readily adopted in the clinic. These developments suggest that the use of integrating viral vectors for introduction of transgenes into therapeutic T cells will be superseded by Cas-induced insertions of templates delivered by either non-integrating viral vectors or naked dsDNA. Continued efforts to enhance the efficiency of Cas-based knockin by means of small molecules or improved design of HDRTs and Cas enzymes are likely to accelerate this transition [120].

The use of unbiased pooled CRISPR knockout screens have shown promise in dissecting cellular processes involved in basic T cell biology including proliferation, differentiation and cell killing. However, introducing thousands of sgRNAs into primary T cells at high complexity is not a straightforward task and only a few studies have been able to do this with high efficiency. In this regard, the recently described Cas12a endonuclease-based screen could provide an alternative platform for performing pooled screens for genome-wide dissections of cellular processes. Cas12a is known to be self-sufficient for multiplexed gene editing as it does not require additional cellular components to process the polycistronic guide precursors. Consequently, the genome-wide library based on Cas12a, called the ‘Mini-human’ library, is a fifth of the size of the libraries used for SpCas9 [121]. Cas12a is also smaller in size than SpCas9 and as discussed previously it has already been shown to be efficient in editing primary T cells [38]. Hence, this smaller-sized Cas12a-based genome-wide library could potentially be highly useful for improving techniques for performing pooled screens in T cells.

The ability to genetically manipulate primary T cells using the CRISPR/Cas9 system has undergone remarkable progress in the last few years, and for researchers entering this field a wide range of options is now available (major techniques summarised in Table 1). Given the therapeutic interest in genetically modified T cells for treatment of diseases such as cancer and autoimmunity, it is likely that this trend will continue, with more techniques emerging for improving the efficiency of primary T cell manipulation while enhancing the precision of editing.

## Figures and Tables

**Table 1:** Summary of all the edit types performed in primary T cells and methods used to deliver Cas enzymes.

		Mode of Delivery						
		Electroporation			Viral			Endogenous
		RNP	mRNA	DNA	Lentivirus	Adenovirus	AAV	Cas9 mice
Knockouts	Single gene	Seki and Rutz 2018 [16] Schumann et al. [20] Vakulskas et al. [23] Hendel et al. [27] Nüssing et al. [36] Shifrut et al. [65]Ye et al. [70] Rupp et al. [84] Gomes-Silva et al. [90]	Ren et al. [21] Hendel et al. [27] Gaudelli et al. [44] Gwiazda et al. [57] Cooper et al. [91]	Hendel et al. [27] Su et al. [30] Mandal et al. [31] Kornete et al. [48] Hou et al. [111] Liu et al. [113]	Legut et al. [79] Singer et al. [86] Kaminski et al. [102] Qi et al. [109]	Li et al. [110] Liu et al. [116]	Wang et al. [115]	LaFleur et al. [34] Chu et al. [35] Dong et al. [69] Ye et al. [70]
	Multiplexed	Webber et al. [26] Nüssing et al. [36] Stadtmauer et al. [81] Yu et al. [114]	Ren et al. [21] Webber et al. [26] Ren et al. [41] Gaudelli et al. [44] Gwiazda et al. [57]	Liu et al. [40] Kornete et al. [48] Liu et al. [113]				
	Pooled	Shifrut et al. [65] Ting et al. [68]						LaFleur et al. [34] Dong et al. [69] Ye et al. [70] Henriksson et al. [71] Cortez et al. [72] Wei et al. [85]
Knockins	Single gene	Schumann et al. [20] Vakulskas 2018 [23] Roth et al. [29] Nüssing et al. [36] Nguyen et al. [56] Wienert et al. [61] Schober et al. [80]	Eyquem et al. [28] Gwiazda et al. [57]	Kornete et al. [48]				
	Mutliplexed	Roth et al. [29] Nguyen et al. [56] Schober et al. [80]	Dai et al. [38]	Kornete et al. [48]				
	Pooled	Roth et al. [73]						

## References

[1] Chaplin D.D. (2010). Overview of the immune response. J. Allergy Clin. Immunol..

[2] Gaud G., Lesourne R., Love P.E. (2018). Regulatory mechanisms in T cell receptor signalling. Nat. Rev. Immunol..

[3] Fuertes Marraco S.A., Neubert N.J., Verdeil G., Speiser D.E. (2015). Inhibitory Receptors Beyond T Cell Exhaustion. Front. Immunol..

[4] Todryk S., Jozwik A., de Havilland J., Hester J. (2019). Emerging Cellular Therapies: T Cells and Beyond. Cells.

[5] Sambi M., Bagheri L., Szewczuk M.R. (2019). Current Challenges in Cancer Immunotherapy: Multimodal Approaches to Improve Efficacy and Patient Response Rates. J. Oncol..

[6] Torikai H., Reik A., Liu P.-Q., Zhou Y., Zhang L., Maiti S., Huls H., Miller J.C., Kebriaei P., Rabinovich B. (2012). A foundation for universal T-cell based immunotherapy: T cells engineered to express a CD19-specific chimeric-antigen-receptor and eliminate expression of endogenous TCR. Blood.

[7] Osborn M.J., Webber B.R., Knipping F., Lonetree C.L., Tennis N., DeFeo A.P., McElroy A.N., Starker C.G., Lee C., Merkel S. (2016). Evaluation of TCR gene editing achieved by TALENs, CRISPR/Cas9, and megaTAL nucleases. Mol. Ther..

[8] Berdien B., Mock U., Atanackovic D., Fehse B. (2014). TALEN-mediated editing of endogenous T-cell receptors facilitates efficient reprogramming of T lymphocytes by lentiviral gene transfer. Gene Ther..

[9] Gaj T., Sirk S.J., Shui S.-L., Liu J. (2016). Genome-Editing Technologies: Principles and Applications. Cold Spring Harb. Perspect. Biol..

[10] Doudna J.A., Charpentier E. (2014). The new frontier of genome engineering with CRISPR-Cas9. Science.

[11] Dominguez A.A., Lim W.A., Qi L.S. (2016). Beyond editing: Repurposing CRISPR-Cas9 for precision genome regulation and interrogation. Nat. Rev. Mol. Cell Biol..

[12] Yeo N.C., Chavez A., Lance-Byrne A., Chan Y., Menn D., Milanova D., Kuo C.C., Guo X., Sharma S., Tung A. (2018). An enhanced CRISPR repressor for targeted mammalian gene regulation. Nat. Methods.

[13] Anzalone A.V., Koblan L.W., Liu D.R. (2020). Genome editing with CRISPR-Cas nucleases, base editors, transposases and prime editors. Nat. Biotechnol..

[14] Pickar-Oliver A., Gersbach C.A. (2019). The next generation of CRISPR–Cas technologies and applications. Nat. Rev. Mol. Cell Biol..

[15] Wang W., Ye C., Liu J., Zhang D., Kimata J.T., Zhou P. (2014). CCR5 gene disruption via lentiviral vectors expressing Cas9 and single guided RNA renders cells resistant to HIV-1 infection. PLoS ONE.

[16] Seki A., Rutz S. (2018). Optimized RNP transfection for highly efficient CRI SPR/Cas9-mediated gene knockout in primary T cells. J. Exp. Med..

[17] Yi G., Choi J.G., Bharaj P., Abraham S., Dang Y., Kafri T., Alozie O., Manjunath M.N., Shankar P. (2014). CCR5 gene editing of resting CD4+ T cells by transient ZFN expression from HIV envelope pseudotyped nonintegrating lentivirus confers HIV-1 resistance in humanized mice. Mol. Therapy Nucleic Acids.

[18] Vella A., Teague T.K., Ihle J., Kappler J., Marrack P. (1997). Interleukin 4 (IL-4) or IL-7 prevents the death of resting T cells: Stat6 is probably not required for the effect of IL-4. J. Exp. Med..

[19] Kishimoto H., Sprent J. (1999). Strong TCR ligation without costimulation causes rapid onset of Fas-dependent apoptosis of naive murine CD4+ T cells. J. Immunol..

[20] Schumann K., Lin S., Boyer E., Simeonov D.R., Subramaniam M., Gate R.E., Haliburton G.E., Ye C.J., Bluestone J.A., Doudna J.A. (2015). Generation of knock-in primary human T cells using Cas9 ribonucleoproteins. Proc. Natl. Acad. Sci. USA.

[21] Ren J., Liu X., Fang C., Jiang S., June C.H., Zhao Y. (2017). Multiplex Genome Editing to Generate Universal CAR T Cells Resistant to PD1 Inhibition. Clin. Cancer Res..

[22] DeWitt M.A., Magis W., Bray N.L., Wang T., Berman J.R., Urbinati F., Heo S.J., Mitros T., Muñoz D.P., Boffelli D. (2016). Selection-free genome editing of the sickle mutation in human adult hematopoietic stem/progenitor cells. Sci. Transl. Med..

[23] Vakulskas C.A., Dever D.P., Rettig G.R., Turk R., Jacobi A.M., Collingwood M.A., Bode N.M., McNeill M.S., Yan S., Camarena J. (2018). A high-fidelity Cas9 mutant delivered as a ribonucleoprotein complex enables efficient gene editing in human hematopoietic stem and progenitor cells. Nat. Med..

[24] Haapaniemi E., Botla S., Persson J., Schmierer B., Taipale J. (2018). CRISPR-Cas9 genome editing induces a p53-mediated DNA damage response. Nat. Med..

[25] Loughran G., Chou M.-Y., Ivanov I.P., Jungreis I., Kellis M., Kiran A.M., Baranov P.V., Atkins J.F. (2014). Evidence of efficient stop codon readthrough in four mammalian genes. Nucleic Acids Res..

[26] Webber B.R., Lonetree C.L., Kluesner M.G., Johnson M.J., Pomeroy E.J., Diers M.D., Lahr W.S., Draper G.M., Slipek N.J., Smeester B.S. (2019). Highly efficient multiplex human T cell engineering without double-strand breaks using Cas9 base editors. Nat. Commun..

[27] Hendel A., Bak R.O., Clark J.T., Kennedy A.B., Ryan D.E., Roy S., Steinfeld I., Lunstad B.D., Kaiser R.J., Wilkens A.B. (2015). Chemically modified guide RNAs enhance CRISPR-Cas genome editing in human primary cells. Nat. Biotechnol..

[28] Eyquem J., Mansilla-Soto J., Giavridis T., Van Der Stegen S.J.C., Hamieh M., Cunanan K.M., Odak A., Gönen M., Sadelain M. (2017). Targeting a CAR to the TRAC locus with CRISPR/Cas9 enhances tumour rejection. Nature.

[29] Roth T.L., Puig-Saus C., Yu R., Shifrut E., Carnevale J., Li P.J., Hiatt J., Saco J., Krystofinski P., Li H. (2018). Reprogramming human T cell function and specificity with non-viral genome targeting. Nature.

[30] Su S., Hu B., Shao J., Shen B., Du J., Du Y., Zhou J., Yu L., Zhang L., Chen F. (2016). CRISPR-Cas9 mediated efficient PD-1 disruption on human primary T cells from cancer patients. Sci. Rep..

[31] Mandal P.K., Ferreira L.M.R., Collins R., Meissner T.B., Boutwell C.L., Friesen M., Vrbanac V., Garrison B.S., Stortchevoi A., Bryder D. (2014). Efficient ablation of genes in human hematopoietic stem and effector cells using CRISPR/Cas9. Cell Stem Cell.

[32] Beil-Wagner J., Dössinger G., Schober K., Vom Berg J., Tresch A., Grandl M., Palle P., Mair F., Gerhard M., Becher B. (2016). T cell-specific inactivation of mouse CD2 by CRISPR/Cas9. Sci. Rep..

[33] Platt R.J., Chen S., Zhou Y., Yim M.J., Swiech L., Kempton H.R., Dahlman J.E., Parnas O., Eisenhaure T.M., Jovanovic M. (2014). CRISPR-Cas9 knockin mice for genome editing and cancer modeling. Cell.

[34] LaFleur M.W., Nguyen T.H., Coxe M.A., Yates K.B., Trombley J.D., Weiss S.A., Brown F.D., Gillis J.E., Coxe D.J., Doench J.G. (2019). A CRISPR-Cas9 delivery system for in vivo screening of genes in the immune system. Nat. Commun..

[35] Chu V.T., Graf R., Wirtz T., Weber T., Favret J., Li X., Petsch K., Tran N.T., Sieweke M.H., Berek C. (2016). Efficient CRISPR-mediated mutagenesis in primary immune cells using CrispRGold and a C57BL/6 Cas9 transgenic mouse line. Proc. Natl. Acad. Sci. USA.

[36] Nüssing S., House I.G., Kearney C.J., Chen A.X.Y., Vervoort S.J., Beavis P.A., Oliaro J., Johnstone R.W., Trapani J.A., Parish I.A. (2020). Efficient CRISPR/Cas9 Gene Editing in Uncultured Naive Mouse T Cells for In Vivo Studies. J. Immunol..

[37] Cong L., Ran F.A., Cox D., Lin S., Barretto R., Habib N., Hsu P.D., Wu X., Jiang W., Marraffini L.A. (2013). Multiplex genome engineering using CRISPR/Cas systems. Science.

[38] Dai X., Park J.J., Du Y., Kim H.R., Wang G., Errami Y., Chen S. (2019). One-step generation of modular CAR-T cells with AAV–Cpf1. Nat. Methods.

[39] McCarty N.S., Graham A.E., Studená L., Ledesma-Amaro R. (2020). Multiplexed CRISPR technologies for gene editing and transcriptional regulation. Nat. Commun..

[40] Liu X., Zhang Y., Cheng C., Cheng A.W., Zhang X., Li N., Xia C., Wei X., Liu X., Wang H. (2017). CRISPR-Cas9-mediated multiplex gene editing in CAR-T cells. Cell Res..

[41] Ren J., Zhang X., Liu X., Fang C., Jiang S., June C.H., Zhao Y. (2017). A versatile system for rapid multiplex genome-edited CAR T cell generation. Oncotarget.

[42] Qasim W., Zhan H., Samarasinghe S., Adams S., Amrolia P., Stafford S., Butler K., Rivat C., Wright G., Somana K. (2017). Molecular remission of infant B-ALL after infusion of universal TALEN gene-edited CAR T cells. Sci. Transl. Med..

[43] Poirot L., Philip B., Schiffer-Mannioui C., Le Clerre D., Chion-Sotinel I., Derniame S., Potrel P., Bas C., Lemaire L., Galetto R. (2015). Multiplex Genome-Edited T-cell Manufacturing Platform for “Off-the-Shelf” Adoptive T-cell Immunotherapies. Cancer Res..

[44] Gaudelli N.M., Lam D.K., Rees H.A., Solá-Esteves N.M., Barrera L.A., Born D.A., Edwards A., Gehrke J.M., Lee S.J., Liquori A.J. (2020). Directed evolution of adenine base editors with increased activity and therapeutic application. Nat. Biotechnol..

[45] Lee H.K., Smith H.E., Liu C., Willi M., Hennighausen L. (2020). Cytosine base editor 4 but not adenine base editor generates off-target mutations in mouse embryos. Commun. Biol..

[46] Zuo E., Sun Y., Wei W., Yuan T., Ying W., Sun H., Yuan L., Steinmetz L.M., Li Y., Yang H. (2019). Cytosine base editor generates substantial off-target single-nucleotide variants in mouse embryos. Science.

[47] Jin S., Zong Y., Gao Q., Zhu Z., Wang Y., Qin P., Liang C., Wang D., Qiu J.-L., Zhang F. (2019). Cytosine, but not adenine, base editors induce genome-wide off-target mutations in rice. Science.

[48] Kornete M., Marone R., Jeker L.T. (2018). Highly Efficient and Versatile Plasmid-Based Gene Editing in Primary T Cells. J. Immunol..

[49] Xu C.L., Ruan M.Z.C., Mahajan V.B., Tsang S.H. (2019). Viral delivery systems for crispr. Viruses.

[50] Li K., Wang G., Andersen T., Zhou P., Pu W.T. (2014). Optimization of genome engineering approaches with the CRISPR/Cas9 system. PLoS ONE.

[51] Boutin S., Monteilhet V., Veron P., Leborgne C., Benveniste O., Montus M.F., Masurier C. (2010). Prevalence of serum IgG and neutralizing factors against adeno-associated virus (AAV) types 1, 2, 5, 6, 8, and 9 in the healthy population: Implications for gene therapy using AAV vectors. Hum. Gene Ther..

[52] Herzog R.W. (2015). Hemophilia Gene Therapy: Caught between a Cure and an Immune Response. Mol. Therapy.

[53] Mingozzi F., High K.A. (2013). Immune responses to AAV vectors: Overcoming barriers to successful gene therapy. Blood.

[54] Moreno-Mateos M.A., Fernandez J.P., Rouet R., Vejnar C.E., Lane M.A., Mis E., Khokha M.K., Doudna J.A., Giraldez A.J. (2017). CRISPR-Cpf1 mediates efficient homology-directed repair and temperature-controlled genome editing. Nat. Commun..

[55] Zetsche B., Gootenberg J.S., Abudayyeh O.O., Slaymaker I.M., Makarova K.S., Essletzbichler P., Volz S.E., Joung J., Van Der Oost J., Regev A. (2015). Cpf1 Is a Single RNA-Guided Endonuclease of a Class 2 CRISPR-Cas System. Cell.

[56] Nguyen D.N., Roth T.L., Li P.J., Chen P.A., Apathy R., Mamedov M.R., Vo L.T., Tobin V.R., Goodman D., Shifrut E. (2020). Polymer-stabilized Cas9 nanoparticles and modified repair templates increase genome editing efficiency. Nat. Biotechnol..

[57] Gwiazda K.S., Grier A.E., Sahni J., Burleigh S.M., Martin U., Yang J.G., Popp N.A., Krutein M.C., Khan I.F., Jacoby K. (2016). High efficiency CRISPR/Cas9-mediated gene editing in primary human T-cells using mutant adenoviral E4orf6/E1b55k “Helper” proteins. Mol. Ther..

[58] Chu V.T., Weber T., Wefers B., Wurst W., Sander S., Rajewsky K., Kühn R. (2015). Increasing the efficiency of homology-directed repair for CRISPR-Cas9-induced precise gene editing in mammalian cells. Nat. Biotechnol..

[59] Yu C., Liu Y., Ma T., Liu K., Xu S., Zhang Y., Liu H., La Russa M., Xie M., Ding S. (2015). Small molecules enhance crispr genome editing in pluripotent stem cells. Cell Stem Cell.

[60] Maruyama T., Dougan S.K., Truttmann M.C., Bilate A.M., Ingram J.R., Ploegh H.L. (2015). Increasing the efficiency of precise genome editing with CRISPR-Cas9 by inhibition of nonhomologous end joining. Nat. Biotechnol..

[61] Wienert B., Nguyen D.N., Guenther A., Feng S.J., Locke M.N., Wyman S.K., Shin J., Kazane K.R., Gregory G.L., Carter M.A.M. (2020). Timed inhibition of CDC7 increases CRISPR-Cas9 mediated templated repair. Nat. Commun..

[62] Chen R., Bélanger S., Frederick M.A., Li B., Johnston R.J., Xiao N., Liu Y.C., Sharma S., Peters B., Rao A. (2014). In vivo RNA interference screens identify regulators of antiviral CD4+ and CD8+ T cell differentiation. Immunity.

[63] Zhou P., Shaffer D.R., Alvarez Arias D.A., Nakazaki Y., Pos W., Torres A.J., Cremasco V., Dougan S.K., Cowley G.S., Elpek K. (2014). In vivo discovery of immunotherapy targets in the tumour microenvironment. Nature.

[64] Hanna R.E., Doench J.G. (2020). Design and analysis of CRISPR–Cas experiments. Nat. Biotechnol..

[65] Shifrut E., Carnevale J., Tobin V., Roth T.L., Woo J.M., Bui C.T., Li P.J., Diolaiti M.E., Ashworth A., Marson A. (2018). Genome-wide CRISPR Screens in Primary Human T Cells Reveal Key Regulators of Immune Function. Cell.

[66] Datlinger P., Rendeiro A.F., Schmidl C., Krausgruber T., Traxler P., Klughammer J., Schuster L.C., Kuchler A., Alpar D., Bock C. (2017). Pooled CRISPR screening with single-cell transcriptome readout. Nat. Methods.

[67] Dixit A., Parnas O., Li B., Chen J., Fulco C.P., Jerby-Arnon L., Marjanovic N.D., Dionne D., Burks T., Raychowdhury R. (2016). Perturb-Seq: Dissecting Molecular Circuits with Scalable Single-Cell RNA Profiling of Pooled Genetic Screens. Cell.

[68] Ting P.Y., Parker A.E., Lee J.S., Trussell C., Sharif O., Luna F., Federe G., Barnes S.W., Walker J.R., Vance J. (2018). Guide Swap enables genome-scale pooled CRISPR-Cas9 screening in human primary cells. Nat. Methods.

[69] Dong M.B., Wang G., Chow R.D., Ye L., Zhu L., Dai X., Park J.J., Kim H.R., Errami Y., Guzman C.D. (2019). Systematic Immunotherapy Target Discovery Using Genome-Scale In Vivo CRISPR Screens in CD8 T Cells. Cell.

[70] Ye L., Park J.J., Dong M.B., Yang Q., Chow R.D., Peng L., Du Y., Guo J., Dai X., Wang G. (2019). In vivo CRISPR screening in CD8 T cells with AAV–Sleeping Beauty hybrid vectors identifies membrane targets for improving immunotherapy for glioblastoma. Nat. Biotechnol..

[71] Henriksson J., Chen X., Gomes T., Ullah U., Meyer K.B., Miragaia R., Duddy G., Pramanik J., Yusa K., Lahesmaa R. (2019). Genome-wide CRISPR Screens in T Helper Cells Reveal Pervasive Crosstalk between Activation and Differentiation. Cell.

[72] Cortez J.T., Montauti E., Shifrut E., Gatchalian J., Zhang Y., Shaked O., Xu Y., Roth T.L., Simeonov D.R., Zhang Y. (2020). CRISPR screen in regulatory T cells reveals modulators of Foxp3. Nature.

[73] Roth T.L., Li P.J., Blaeschke F., Nies J.F., Apathy R., Mowery C., Yu R., Nguyen M.L.T., Lee Y., Truong A. (2020). Pooled Knockin Targeting for Genome Engineering of Cellular Immunotherapies. Cell.

[74] Xia A., Zhang Y., Xu J., Yin T., Lu X.-J. (2019). T Cell Dysfunction in Cancer Immunity and Immunotherapy. Front. Immunol..

[75] Bailey S.R., Maus M.V. (2019). Gene editing for immune cell therapies. Nat. Biotechnol..

[76] Cornu T.I., Mussolino C., Cathomen T. (2017). Refining strategies to translate genome editing to the clinic. Nat. Med..

[77] Porteus M.H. (2019). A new class of medicines through DNA editing. N. Engl. J. Med..

[78] Gallegos A.M., Xiong H., Leiner I.M., Sušac B., Glickman M.S., Pamer E.G., Van Heijst J.W.J. (2016). Control of T cell antigen reactivity via programmed TCR downregulation. Nat. Immunol..

[79] Legut M., Dolton G., Mian A.A., Ottmann O.G., Sewell A.K. (2018). CRISPR-mediated TCR replacement generates superior anticancer transgenic t cells. Blood.

[80] Schober K., Müller T.R., Gökmen F., Grassmann S., Effenberger M., Poltorak M., Stemberger C., Schumann K., Roth T.L., Marson A. (2019). Orthotopic replacement of T-cell receptor α- and β-chains with preservation of near-physiological T-cell function. Nat. Biomed. Eng..

[81] Stadtmauer E.A., Fraietta J.A., Davis M.M., Cohen A.D., Weber K.L., Lancaster E., Mangan P.A., Kulikovskaya I., Gupta M., Chen F. (2020). CRISPR-engineered T cells in patients with refractory cancer. Science.

[82] Charlesworth C.T., Deshpande P.S., Dever D.P., Camarena J., Lemgart V.T., Cromer M.K., Vakulskas C.A., Collingwood M.A., Zhang L., Bode N.M. (2019). Identification of preexisting adaptive immunity to Cas9 proteins in humans. Nat. Med..

[83] Martins F., Sofiya L., Sykiotis G.P., Lamine F., Maillard M., Fraga M., Shabafrouz K., Ribi C., Cairoli A., Guex-Crosier Y. (2019). Adverse effects of immune-checkpoint inhibitors: Epidemiology, management and surveillance. Nat. Rev. Clin. Oncol..

[84] Rupp L.J., Schumann K., Roybal K.T., Gate R.E., Ye C.J., Lim W.A., Marson A. (2017). CRISPR/Cas9-mediated PD-1 disruption enhances anti-Tumor efficacy of human chimeric antigen receptor T cells. Sci. Rep..

[85] Wei J., Long L., Zheng W., Dhungana Y., Lim S.A., Guy C., Wang Y., Wang Y.-D., Qian C., Xu B. (2019). Targeting REGNASE-1 programs long-lived effector T cells for cancer therapy. Nature.

[86] Singer M., Wang C., Cong L., Marjanovic N.D., Kowalczyk M.S., Zhang H., Nyman J., Sakuishi K., Kurtulus S., Gennert D. (2016). A Distinct Gene Module for Dysfunction Uncoupled from Activation in Tumor-Infiltrating T Cells. Cell.

[87] Campana D., Van Dongen J.J.M., Mehta A., Coustan-Smith E., Wolvers-Tettero I.L.M., Ganeshaguru K., Janossy G. (1991). Stages of T-cell Receptor Protein Expression in T-cell Acute Lymphoblastic Leukemia. Blood.

[88] Jones N.H., Clabby M.L., Dialynas D.P., Huang H.J.S., Herzenberg L.A., Strominger J.L. (1986). Isolation of complementary DNA clones encoding the human lymphocyte glycoprotein T1/Leu-1. Nature.

[89] Pui C.-H., Behm F.G., Crist W.M. (1993). Clinical and Biologic Relevance of Immunologic Marker Studies in Childhood Acute Lymphoblastic Leukemia. Blood.

[90] Gomes-Silva D., Srinivasan M., Sharma S., Lee C.M., Wagner D.L., Davis T.H., Rouce R.H., Bao G., Brenner M.K., Mamonkin M. (2017). CD7-edited T cells expressing a CD7-specific CAR for the therapy of T-cell malignancies. Blood.

[91] Cooper M.L., Choi J., Staser K., Ritchey J.K., Devenport J.M., Eckardt K., Rettig M.P., Wang B., Eissenberg L.G., Ghobadi A. (2018). An “off-the-shelf” fratricide-resistant CAR-T for the treatment of T cell hematologic malignancies. Leukemia.

[92] Mamonkin M., Rouce R.H., Tashiro H., Brenner M.K. (2015). A T-cell-directed chimeric antigen receptor for the selective treatment of T-cell malignancies. Blood.

[93] Depil S., Duchateau P., Grupp S.A., Mufti G., Poirot L. (2020). “Off-the-shelf” allogeneic CAR T cells: Development and challenges. Nat. Rev. Drug Discov..

[94] Aversa F., Tabilio A., Velardi A., Cunningham I., Terenzi A., Falzetti F., Ruggeri L., Barbabietola G., Aristei C., Latini P. (1998). Treatment of high-risk acute leukemia with T-cell-depleted stem cells from related donors with one fully mismatched HLA haplotype. N. Engl. J. Med..

[95] Abdelhakim H., Abdel-Azim H., Saad A. (2017). Role of αβ T Cell Depletion in Prevention of Graft versus Host Disease. Biomedicines.

[96] Felix N.J., Allen P.M. (2007). Specificity of T-cell alloreactivity. Nat. Rev. Immunol..

[97] Wang D., Quan Y., Yan Q., Morales J.E., Wetsel R.A. (2015). Targeted Disruption of the β2-Microglobulin Gene Minimizes the Immunogenicity of Human Embryonic Stem Cells. Stem Cells Transl. Med..

[98] Xiao Q., Guo D., Chen S. (2019). Application of CRISPR/Cas9-based gene editing in HIV-1/AIDS therapy. Front. Cell. Infect. Microbiol..

[99] Chun T.W., Moir S., Fauci A.S. (2015). HIV reservoirs as obstacles and opportunities for an HIV cure. Nat. Immunol..

[100] Ebina H., Misawa N., Kanemura Y., Koyanagi Y. (2013). Harnessing the CRISPR/Cas9 system to disrupt latent HIV-1 provirus. Sci. Rep..

[101] Hu W., Kaminski R., Yang F., Zhang Y., Cosentino L., Li F., Luo B., Alvarez-Carbonell D., Garcia-Mesa Y., Karn J. (2014). RNA-directed gene editing specifically eradicates latent and prevents new HIV-1 infection. Proc. Natl. Acad. Sci. USA.

[102] Kaminski R., Chen Y., Fischer T., Tedaldi E., Napoli A., Zhang Y., Karn J., Hu W., Khalili K. (2016). Elimination of HIV-1 Genomes from Human T-lymphoid Cells by CRISPR/Cas9 Gene Editing. Sci. Rep..

[103] Liao H.K., Gu Y., Diaz A., Marlett J., Takahashi Y., Li M., Suzuki K., Xu R., Hishida T., Chang C.J. (2015). Use of the CRISPR/Cas9 system as an intracellular defense against HIV-1 infection in human cells. Nat. Commun..

[104] Kaminski R., Bella R., Yin C., Otte J., Ferrante P., Gendelman H.E., Li H., Booze R., Gordon J., Hu W. (2016). Excision of HIV-1 DNA by gene editing: A proof-of-concept in vivo study. Gene Ther..

[105] Yin L., Hu S., Mei S., Sun H., Xu F., Li J., Zhu W., Liu X., Zhao F., Zhang D. (2018). CRISPR/Cas9 Inhibits Multiple Steps of HIV-1 Infection. Hum. Gene Ther..

[106] Dash P.K., Kaminski R., Bella R., Su H., Mathews S., Ahooyi T.M., Chen C., Mancuso P., Sariyer R., Ferrante P. (2019). Sequential LASER ART and CRISPR Treatments Eliminate HIV-1 in a Subset of Infected Humanized Mice. Nat. Commun..

[107] Hütter G., Nowak D., Mossner M., Ganepola S., Müßig A., Allers K., Schneider T., Hofmann J., Kücherer C., Blau O. (2009). Long-Term Control of HIV by CCR5 Delta32/Delta32 Stem-Cell Transplantation. N. Engl. J. Med..

[108] Tebas P., Stein D., Tang W.W., Frank I., Wang S.Q., Lee G., Spratt S.K., Surosky R.T., Giedlin M.A., Nichol G. (2014). Gene Editing of CCR5 in Autologous CD4 T Cells of Persons Infected with HIV. N. Engl. J. Med..

[109] Qi C., Li D., Jiang X., Jia X., Lu L., Wang Y., Sun J., Shao Y., Wei M. (2018). Inducing CCR5Δ32/Δ32 Homozygotes in the Human Jurkat CD4+ Cell Line and Primary CD4+ Cells by CRISPR-Cas9 Genome-Editing Technology. Mol. Ther. Nucleic Acids.

[110] Li C., Guan X., Du T., Jin W., Wu B., Liu Y., Wang P., Hu B., Griffin G.E., Shattock R.J. (2015). Inhibition of HIV-1 infection of primary CD4+ T-cells by gene editing of CCR5 using adenovirus-delivered CRISPR/Cas9. J. Gen. Virol..

[111] Hou P., Chen S., Wang S., Yu X., Chen Y., Jiang M., Zhuang K., Ho W., Hou W., Huang J. (2015). Genome editing of CXCR4 by CRISPR/cas9 confers cells resistant to HIV-1 infection. Sci. Rep..

[112] Connor R.I., Sheridan K.E., Ceradini D., Choe S., Landau N.R. (1997). Change in coreceptor use correlates with disease progression in HIV-1- infected individuals. J. Exp. Med..

[113] Liu Z., Chen S., Jin X., Wang Q., Yang K., Li C., Xiao Q., Hou P., Liu S., Wu S. (2017). Genome editing of the HIV co-receptors CCR5 and CXCR4 by CRISPR-Cas9 protects CD4+ T cells from HIV-1 infection. Cell Biosci..

[114] Yu S., Yao Y., Xiao H., Li J., Liu Q., Yang Y., Adah D., Lu J., Zhao S., Qin L. (2018). Simultaneous Knockout of CXCR4 and CCR5 Genes in CD4+ T Cells via CRISPR/Cas9 Confers Resistance to Both X4- and R5-Tropic Human Immunodeficiency Virus Type 1 Infection. Hum. Gene Ther..

[115] Wang Q., Chen S., Xiao Q., Liu Z., Liu S., Hou P., Zhou L., Hou W., Ho W., Li C. (2017). Genome modification of CXCR4 by Staphylococcus aureus Cas9 renders cells resistance to HIV-1 infection. Retrovirology.

[116] Liu Z., Liang J., Chen S., Wang K., Liu X., Liu B., Xia Y., Guo M., Zhang X., Sun G. (2020). Genome editing of CCR5 by AsCpf1 renders CD4+T cells resistance to HIV-1 infection. Cell Biosci..

[117] Tian S., Choi W.-T., Liu D., Pesavento J., Wang Y., An J., Sodroski J.G., Huang Z. (2005). Distinct Functional Sites for Human Immunodeficiency Virus Type 1 and Stromal Cell-Derived Factor 1α on CXCR4 Transmembrane Helical Domains. J. Virol..

[118] DiTommaso T., Cole J.M., Cassereau L., Buggé J.A., Sikora Hanson J.L., Bridgen D.T., Stokes B.D., Loughhead S.M., Beutel B.A., Gilbert J.B. (2018). Cell engineering with microfluidic squeezing preserves functionality of primary immune cells in vivo. Proc. Natl. Acad. Sci. USA.

[119] Anzalone A.V., Randolph P.B., Davis J.R., Sousa A.A., Koblan L.W., Levy J.M., Chen P.J., Wilson C., Newby G.A., Raguram A. (2019). Search-and-replace genome editing without double-strand breaks or donor DNA. Nature.

[120] Liu M., Rehman S., Tang X., Gu K., Fan Q., Chen D., Ma W. (2018). Methodologies for Improving HDR Efficiency. Front. Genet..

[121] Liu J., Srinivasan S., Li C.-Y., Ho I.-L., Rose J., Shaheen M., Wang G., Yao W., Deem A., Bristow C. (2019). Pooled library screening with multiplexed Cpf1 library. Nat. Commun..

